# Polymers as Efficient Non-Viral Gene Delivery Vectors: The Role of the Chemical and Physical Architecture of Macromolecules

**DOI:** 10.3390/polym16182629

**Published:** 2024-09-18

**Authors:** Majad Khan

**Affiliations:** 1 Department of Chemistry, King Fahd University of Petroleum & Minerals KFUPM, Dahran 31261, Saudi Arabia; majad.khan@kfupm.edu.sa; Tel.: +966-0138601565; 2 Interdisciplinary Research Center for Hydrogen Technologies and Carbon Management (IRC-HTCM), King Fahd University of Petroleum & Minerals KFUPM, Dahran 31261, Saudi Arabia; 3 Interdisciplinary Research Center for Refining and Advanced Chemicals (IRC-CRAC), King Fahd University of Petroleum & Minerals (KFUPM), Dhahran 31261, Saudi Arabia

**Keywords:** gene delivery, non-viral vectors, DNA complexation, poly-L-lysine, poly(ethylene imine), dendrimer, linear polymers, star polymers, hyperbranched polymers, brush polymers, comb polymers, hydrogels, natural polymers

## Abstract

Gene therapy is the technique of inserting foreign genetic elements into host cells to achieve a therapeutic effect. Although gene therapy was initially formulated as a potential remedy for specific genetic problems, it currently offers solutions for many diseases with varying inheritance patterns and acquired diseases. There are two major groups of vectors for gene therapy: viral vector gene therapy and non-viral vector gene therapy. This review examines the role of a macromolecule’s chemical and physical architecture in non-viral gene delivery, including their design and synthesis. Polymers can boost circulation, improve delivery, and control cargo release through various methods. The prominent examples discussed include poly-L-lysine, polyethyleneimine, comb polymers, brush polymers, and star polymers, as well as hydrogels and natural polymers and their modifications. While significant progress has been made, challenges still exist in gene stabilization, targeting specificity, and cellular uptake. Overcoming cytotoxicity, improving delivery efficiency, and utilizing natural polymers and hybrid systems are vital factors for prospects. This comprehensive review provides an illuminating overview of the field, guiding the way toward innovative non-viral-based gene delivery solutions.

## 1. Introduction and Background to Gene Delivery

Gene therapy is a promising field in personalized medicine thanks to its efficient molecular mechanisms combating diseases. The use of viral vectors, the most common delivery vehicle currently employed in clinical trials, allows for the precise delivery of therapeutic genetic material to cells [1]. Gene therapy is an emerging science that uses genes to prevent or treat infections, allowing medical practitioners to address diseases by inserting genes into patients’ cells rather than using drugs or surgery. Researchers are investigating a variety of gene therapy procedures, including replacing mutant genes with healthy ones, deactivating faulty genes, and introducing new genes to protect against disease. Therapeutic genetic agents must enter host cell nuclei to cause gene expression [2]. Gene therapy offers hope for treating incurable diseases [3], but the chemical fragility of therapeutic genes limits its applicability. Naked genes lose bioactivity quickly due to serum nuclease degradation [4]. Since therapeutic genes possess a negative charge and are hydrophilic, this, coupled with the fact that they are most often of high molecular weight, results in poor membrane permeability, low cellular uptake, and limited blood circulation stability. Gene therapy faces numerous extracellular and intracellular barriers, including rapid clearance, the immune response, lack of tissue specificity, endosomal escape issues, and offloading of gene cargo from delivery vectors [3]. Gene therapy provides somatic cells with genetic information to produce therapeutic proteins, requiring an effective delivery system that includes a plasmid-based gene expression system, a gene encoding the therapeutic protein, and a stable mechanism to deliver the plasmid to target cells [5]. Gene delivery vectors are crucial in overcoming these obstacles by transporting inheritable factors to targeted cells, facilitating intracellular unpacking, and ensuring successful transfection processes [6]. Using appropriate gene delivery vectors maximizes the transfection efficiency of therapeutic genes while minimizing adverse effects in patients. These vectors are essential for the effective application of gene therapy. In achieving effective gene delivery, vectors must ensure efficient cellular entry, endosomal escape, cytoplasmic trafficking, and offloading of genetic material without eliciting immunogenic or cytotoxic responses [7,8]. Viral vectors like retroviruses and adenoviruses are used in 70% of gene therapy trials. Despite high efficiency, they pose safety risks, have low gene capacity, and are difficult to prepare [9]. Viral vectors, first used in the 1980s, employed the vaccinia virus to protect chimpanzees from Hepatitis B. Non-viral delivery systems were also developed to induce phenotypic changes via DNA exposure [10]. Non-viral vectors like lipid nanoparticles and cationic polymers offer high gene loading capacity, safety, simplicity, and practicality, showing significant potential for further clinical development and application [11]. Ideal non-viral vectors for gene therapy should be therapeutic systems with low toxicity, efficient DNA complexation, membrane penetration ability, and intracellular plasmid release capability.

The genetic manipulation of animals has significantly transformed the in vivo modeling of human diseases. Mice and rats are frequently subjected to germline mutations, ensuring that gene modifications are present in all cells. These genetically modified animal models play a vital role in preclinical research across various diseases [12]. However, it is now acknowledged that many disorders originate from somatic mutations during early embryonic development, resulting in tissue mosaicism and impacting multiple organ regions. The specific gene, nature, and timing of these mutations determine the severity of the phenotype. Within the field of neuroscience, studies have demonstrated a correlation between somatic mutations during brain development and neurological conditions, such as cortical malformations and brain tumors. These conditions are often associated with epilepsy [13,14]. Gene delivery has been explored in various real-life cases involving both humans and animals. Non-viral methods are particularly desirable due to their reduced risk of immune responses and insertional mutagenesis. Some examples include the following: Electroporation is the most frequently utilized non-viral gene delivery technology because of its precise dose control, diversity in suitable varieties of cells, and high effectiveness of transfection and survival of cell levels [15]. Electroporation, initially identified by Neumann et al. in 1982, allows for the intracellular transport of genetic material by sending electrical impulses to cells. This electrical impulse causes a potential differential within the cell membrane, breaking the lipid bilayer and creating transitory holes called aqueous routes [16].

Frangoul et al. [17] sed MaxCyte GTx to electroporate the CD34^+^ hematopoietic stem and progenitor cells from healthy donors with CRISPR-Cas9. They modified the BCL11A gene to create an indel, which represses fetal hemoglobin. Two patients, one with sickle cell disease and one with transfusion-dependent β-thalassemia, received a single CTX001 infusion and improved their conditions [17]. Electroporation can also be performed in living organisms using methods like genome editing via oviductal nucleic acid (NA) delivery (GONAD). Takahashi et al. developed GONAD, a technique for germline genome editing via oviductal nucleic acid delivery and in situ electroporation in pregnant mice, achieving successful editing at the two-cell embryo stage [18,19]. Liposomes, recognized in the 1980s for drug delivery due to their biocompatibility and size flexibility, led to the global approval of liposome-based drugs, paving the way for developing lipid nanoparticles (LNPs) as gene delivery systems [20]. The Food and Drug Agency (FDA) has approved liposomal formulations of drugs like doxorubicin for treating cancers such as Kaposi’s sarcoma, breast cancer, ovarian cancer, and multiple myelomas. Cationic liposomes transfect cultured cells via pH-triggered fusion and membrane content release [21]. Francia et al. showed that tailoring LNP properties, such as size and charge, can improve gene delivery specificity and efficacy. Cationic lipid composition can alter biodistribution, shifting from the liver to the spleen and lungs and enhancing delivery to target sites [22]. This review aims to provide a comprehensive overview of macromolecules’ chemical and physical architecture in non-viral gene delivery, explicitly focusing on polymeric systems. It will explore various types of macromolecules utilized, discussing their performance, toxicity, and the challenges encountered in gene delivery. It will also explore the prospects for polymers in non-viral gene delivery, including potential solutions to enhance delivery efficiency and overcome cytotoxicity. Furthermore, it will touch on utilizing natural polymers and hybrid systems. Ultimately, the goal is to present a thorough understanding of the role of polymers in non-viral gene delivery and their potential for future advancements.

## 2. Introduction and Background to Non-Viral Systems

**Figure 1:**
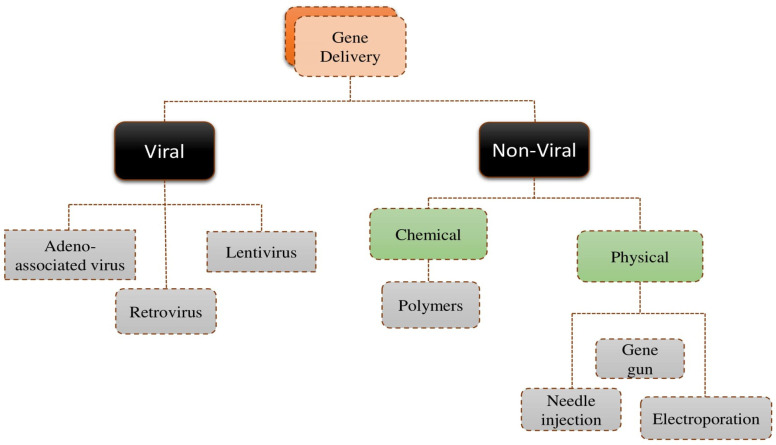
Classification of common gene delivery methods [23].

Gene delivery is categorized into two distinct groups: non-viral and viral, where non-viral is further subdivided into chemical and physical, as shown in Figure 1 below. Chemical and physical techniques are referred to as transfection technologies. Adeno-associated virus, herpesvirus, and lentivirus are examples of viruses utilized for viral gene delivery. Non-viral gene delivery techniques can be classified as chemical or physical. Chemical approaches include polymers and lipids, whereas physical methods use physical qualities and pressures to carry genetic code into cells. Biological strategies are also known as transduction technologies. Biological approaches of direct injection of DNAs and RNAs are based on hereditarily designed diseases to transfer non-viral DNAs into cells by viral delivery. This method targets hard-to-transfect cell types for protein overexpression or knockdown [23]. Numerous biological obstacles, such as extracellular matrix trapping, cell membrane piercing, endosomal escape, and nuclear transport, affect gene delivery. Chemical–physical structures that improve stability, absorption by cells, and endosomal escape—such as cationic polymers, PEGylation, and the surface modifications of nanocarriers—assist in getting past these obstacles. Future approaches may concentrate on stimuli-responsive substances that adjust to cellular conditions, maximize delivery, and reduce off-target effects to achieve more efficient gene therapy.

**Figure 2:**
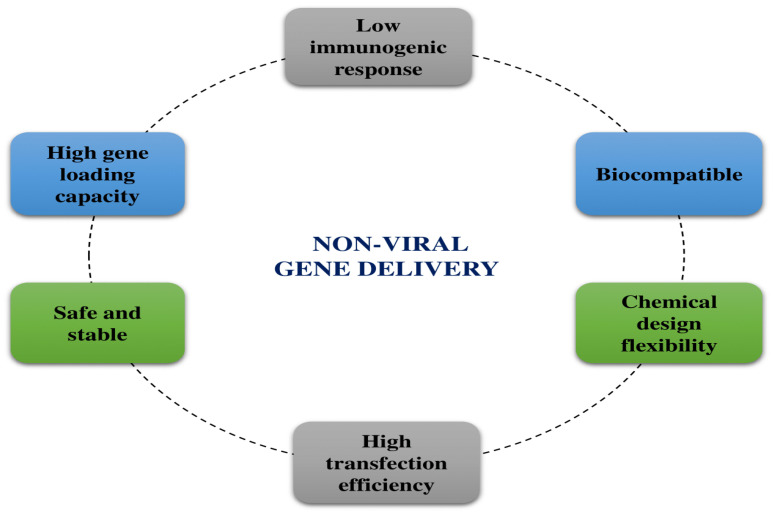
Benefits of non-viral gene delivery systems.

Non-viral gene delivery, otherwise known as transfection, involves introducing exogenous nucleic acids into target cells [24]. It is pivotal in nanomedicine for genetic-level disease prevention and treatment in nano-biotechnology for recombinant protein production [25]. Non-viral vectors have gained significant attention over the past two decades due to their advantages, such as low immunogenicity, safety, high gene loading capacity, stability, and flexible chemical design, as shown in Figure 2 [11].

**Figure 3:**
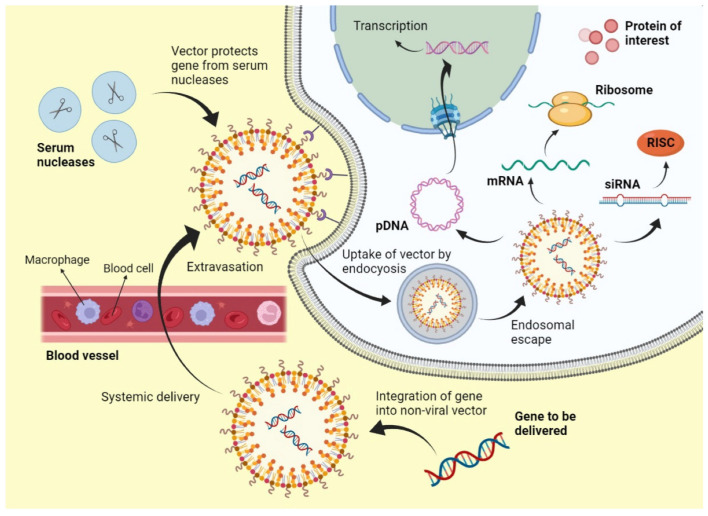
Mechanism of non-viral gene delivery systems [29].

Non-viral gene delivery, large-scale production, and chemical modification are relatively straightforward. Crucially, non-viral vector systems are not limited by the gene size to be delivered [26,27]. Non-viral gene delivery vectors are categorized into physical methods—electroporation, sonoporation, magnetoporation, microinjection, needle injection, gene gun—and chemical systems, including polymeric materials. Most chemical systems are cationic, allowing them to bind with negatively charged DNA through electrostatic interactions, forming positively charged complexes. These complexes bind to negatively charged cellular membranes and internalize into cells. Once inside, they must escape endosomal and lysosomal degradation to deliver the transgene to the nucleus (for DNA) or cytoplasm (for mRNA) [28]. Figure 3 below demonstrates the mechanism of non-viral gene delivery [29].

Polymeric gene delivery systems employ biocompatible polymers to encapsulate genetic material primarily via electrostatic complexation (DNA or RNA), preserve it from degradation, and enhance cellular uptake. These systems provide design versatility, scalability, and reduced immunogenicity. Over the past two decades, significant efforts have focused on creating advanced tools and materials for nucleic acid delivery into cells [30]. Cationic lipids and polymers, known as transfection reagents, are widely used due to their effectiveness and ease of use. They form lipoplexes and polyplexes with anionic NAs, facilitating intracellular delivery [31]. Gene delivery has been transformed by mRNA technology, which offers a flexible platform for quick development and tailored treatments [32]. In some aspects, it is an ideal regimen to treat a variety of illnesses, including genetic problems and infectious diseases, since it can direct cells to manufacture particular proteins. Recent developments in targeted delivery, cellular uptake, and mRNA stability have been greatly enhanced by the use of lipid-based nanoparticles and other nanoparticle-based delivery methods. This has demonstrated the promise of mRNA in customized medicine and pandemic preparation, leading to effective uses in vaccinations and treatments. The current research and development demonstrate the revolutionary effect of mRNA on gene therapy [33]. Liposome and cationic lipid transfection have become among the most commonly used methods for gene delivery, introducing foreign genes into cells. In most gene therapy efforts, the primary goal of using liposomes for transfecting foreign genes is to prevent their toxicity from harming the body. Consequently, factors such as the liposome-to-plasmid ratio, cell density, transfection time, and serum level in the medium all affect transfection efficiency. To improve transfection efficiency and safety, ongoing research is necessary to explore optimal transfection conditions [34]. The development of polymer systems for gene delivery has been slower than lipid systems due to the complexity of polymer design, synthesis, and optimization. Polymers often face challenges with biocompatibility and toxicity, requiring extensive modifications to ensure safety and efficacy. In contrast, lipid systems benefit from established, proven technologies and simpler formulation processes. Despite these hurdles, ongoing research aims to address polymer limitations and enhance their development pace.

Polymer-based drug delivery systems (DDSs) have received considerable attention for their ability to target tumor tissues and enhance therapeutic efficacy. FDA-approved biodegradable polymer poly(L-lactide-co-glycolide) (PLGA) has led to clinically approved formulations like Decapeptyl, Suprecur MP, and Lupron Depot [35]. Polymer-based drug delivery systems (DDSs) enhance therapeutic bioavailability, safety, and efficacy by regulating drug release rates targeting specific biological sites. Utilizing biodegradable, bio-absorbable polymers, these systems offer a safe and effective strategy to overcome chemotherapy limitations in cancer treatment [36]. An effective non-viral systemic gene vector was created by covering the polyplex micelle with a hydrophobic layer between poly(ethylene glycol) shells and complexed pDNA cores. The hydrophobic layer was created by easily complexing pDNA with mixed block copolymers [37]. Polymeric systems show capacity for non-viral gene delivery, with cationic polymers forming DNA complexes and facilitating cellular delivery. These polymers have been extensively studied for their potential in gene delivery applications.

## 3. Macromolecules Used in Gene Delivery

**Figure 4:**
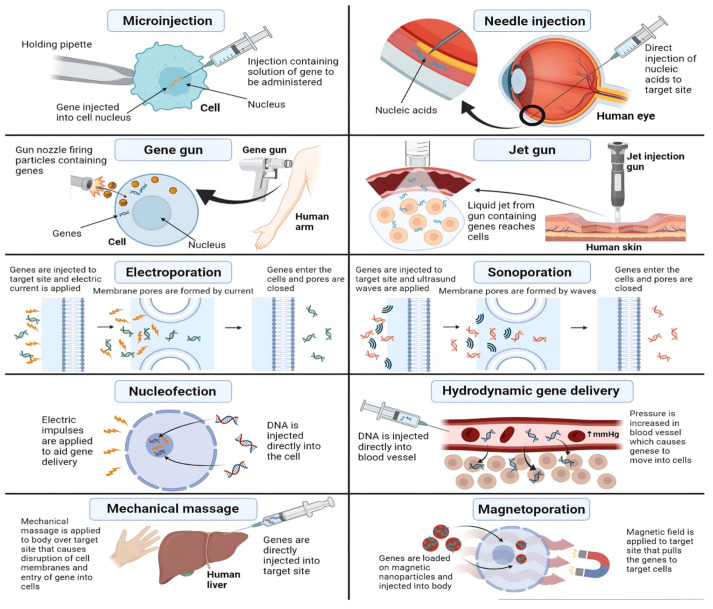
Physical methods for non-viral gene delivery [43].

Over the past few decades, biotechnological developments have driven the explosive growth of macromolecular drug discovery, which includes DNA, RNA, peptides, and proteins [38]. These bio-macromolecules are used in drug delivery in several ways, such as carrier materials, active pharmaceutical components, and targeting agents. The U.S. FDA categorizes macromolecules into various categories: vaccines, blood products, allergenic extracts, transplantable human tissues, DNA therapy preparations, therapeutic cell preparations, and reagents for detecting infectious agents [39]. Macromolecules as potential therapeutic options have attracted more attention because of their unique affinity, target specificity, and multifaceted functions, especially when treating cancer, a widespread and rapidly rising worldwide health issue [40]. Non-viral gene delivery systems utilize biocompatible materials like lipids, DNA, and cationic polymers, reducing immune response risks. These approaches enable safe, effective gene delivery, leveraging conjugate complexes and plasmids [41]. Physical non-viral gene delivery methods, such as microinjection, gene gun, electroporation, and sonoporation, introduce genetic material into cells, as illustrated in Figure 4 below [42]. Needle injection directly delivers genetic elements, while ballistic DNA injection uses gold-coated DNA particles. Electroporation, sonoporation, and photoporation create membrane pores with electric pulses, sound waves, and laser pulses. Magnetoporation concentrates nucleic acids into target cells using magnetic particles and an external magnetic field, and hydroporation manipulates cell permeability via the hydrodynamic capillary effect.

Non-viral chemical techniques use synthetic or natural substances to generate particles enhancing gene transfer into cells [10,44]. Non-viral chemical vectors enter cells via endocytosis. Two types, liposomes and polymers, facilitate this process. Liposomal vectors form lipoplexes, enhancing gene delivery, while polymer-based vectors create polyplexes through DNA interaction. These approaches efficiently transfer genetic material into cells without viral vectors.

### 3.1. Role of the Chemical and Physical Architecture of Macromolecules for Non-Viral Gene Therapy

Non-viral gene delivery strategies use different macromolecules to convey genetic information to cells. The toxicity, chemical functionality, and physical architecture of these macromolecules differ. This article covers six classes of macromolecules involved in non-viral gene transport. Linear polymers are synthesized via living polymerization methods, such as atom transfer radical polymerization (ATRP) [45], which is a very efficient method for producing well-defined polymers or copolymers having a predefined molecular weight, a narrow molecular weight dispersion, and a high degree of chain end functionality, the controlled/living polymerization process, or reversible addition-fragmentation chain transfer (RAFT) [46]. The controlled/living radical polymerization process enables exact control over the molecular weight, polydispersity, and polymer design, and is functionalized with amine or carboxyl groups to enhance DNA binding for moderate transfection efficiency. Polymers as non-viral vectors are advantageous because they are simple to synthesize, inexpensive, can be designed to be biodegradable, are non-immunogenic, and can be extensively modified [42,43,44,45]. They protect nucleic acid drugs by forming polyelectrolyte complexes. Commonly used polymers in gene therapy include poly-L-lysine (PLL), poly(ethylene imine) (PEI), polyamidoamine (PAMAM), poly-L-lysine-grafted-polyethylene glycol (PLL-g-PEG), hydrogel, and natural polymers. Their characteristics and limitations are detailed in Table 1 below.

#### 3.1.1. Linear Polymers

Linear polymers are a crucial class of materials for non-viral gene delivery, valued for their straightforward structure and ease of functionalization. Below are some commonly used linear polymers in this field.

##### Poly-L-lysine (PLL)

**Figure 5:**
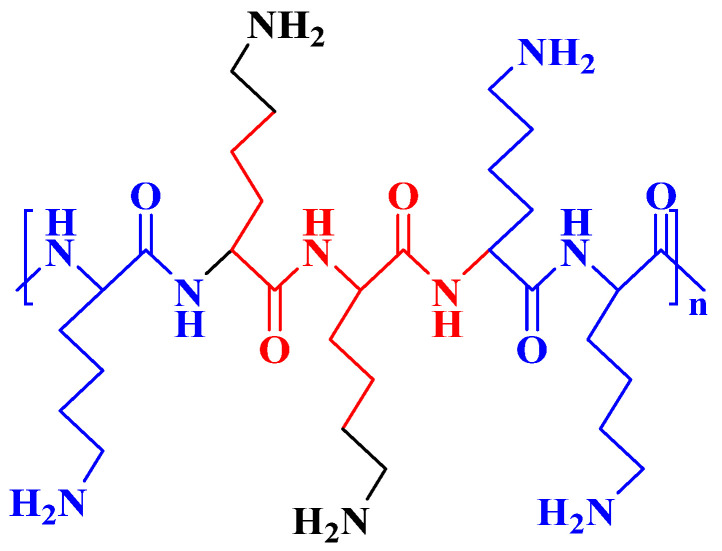
Chemical structure of linear poly-L-lysine (PLL).

Poly-L-lysine (PLL) is a synthetic linear polypeptide made of repeated L-lysine residues. PLL is well-known for its capacity to bind DNA that is negatively charged and form stable complexes for gene delivery, particularly when the molecular weight of the DNA is more than 3000 Da. PLLs are met with difficulties such as complex instability in serum, poor endosomal escape, and cytotoxicity, despite their ease of chemical manipulation and promise for functionalization. These restrictions can be lessened, however, by making changes like PEGylation, which improves stability and decreases toxicity, or by adding reduction- or pH-sensitive groups to increase transfection efficiency and targeting [47]. Both PLL and its low molecular weight analogs, oligolysines, were studied for their ability to condense DNA into nanoparticles and facilitate its delivery to cells [48], as shown in Figure 5.

Nayvelt et al. proposed that DNA can condense into nanoparticles with various shapes, such as toroids, spheroids, cubes, and rods, through the action of oligo- and poly-L-lysine [49]. Korolev et al. identified salt-dependent and salt-independent interaction regimes between oligolysines and plasmid DNA [50]. Modifying PEI with PLL improved transfection efficacy in HeLa cells and decreased toxicity [51]. Malik et al. demonstrated that polylysine-modified PEI can activate genetically engineered mesenchymal stem cells for combinational suicidal gene therapy in glioblastoma, presenting a promising treatment strategy [52]. Kodama et al. developed dendrigraft poly-L-lysine for gene delivery, creating a ternary complex with γ-PGA and DNA that showed high transfection efficiency in various tissues.

In contrast, others synthesize poly-L-lysine copolymers to enhance gene delivery [53,54]. Yu et al. created a copolymer by grafting poly-L-lysine onto chitosan, which improved transfection effectiveness and reduced cytotoxicity in vivo. Combining PLL’s strong DNA binding capabilities with the degradation and compatibility of chitosan produced higher transfection effectiveness than using either polymer alone [55]. PLL, a cationic homopolypeptide with positively charged amino groups at pH 7, forms replica particles (RPs) that co-adsorb plasmid DNA for gene delivery, enabling crosslinked PLL-based gene transfer systems.

##### Linear Poly(ethylene imine) (PEI)

**Figure 6:**
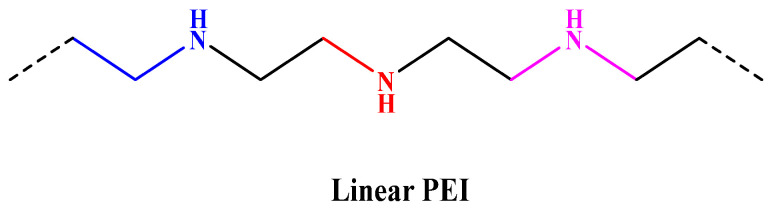
Structure of linear PEI.

Poly(ethylene imine) (PEI) is a cationic polymer composed of secondary amino groups and ethylene units (-NH-CH_2_CH_2_-). It is widely used as a transfection reagent and nanocarriers in drug delivery systems, improving the effectiveness of targeted therapies and gene treatments. While PEI has been extensively studied and utilized as a carrier for gene delivery, its main drawback is its toxicity [56,57,58]. PEI is also known as polyfunctional aziridine, originating from aziridine ring-opening polymerization. Its high positive charge density potential arises from protonated amino groups in the chain on every third atom [58,59]. The linear (CH_2_CH_2_NH)n structure is illustrated below in Figure 6 [60,61].

The diverse PEI compositions provide unique structures with different characteristics and relativities. Zhou et al. developed a modified PEI derivative with reduced cytotoxicity that was obtained by treating PEI with a cyclic amine derivative. This derivative showed promising anti-tumor properties, inhibiting CXCR4 and tumor cell invasion [62].

**Scheme 1:**
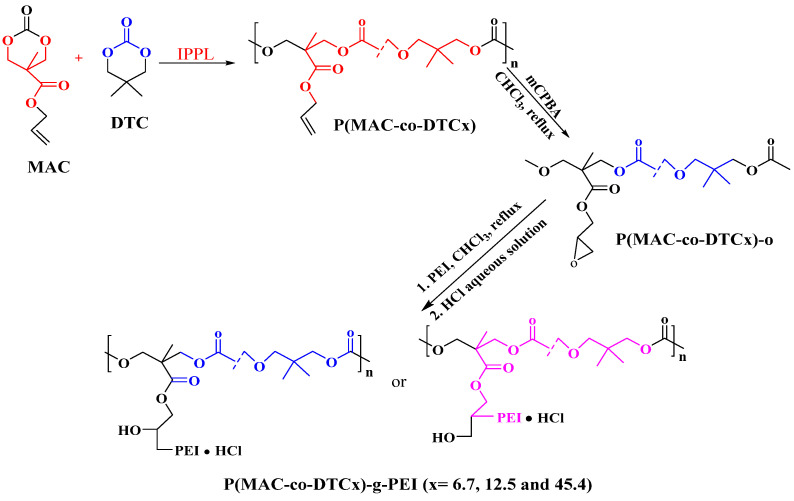
The synthetic approach for P (MAC-co-DTC)-g-PEI copolymers.

Gupta et al. demonstrated enhanced gene delivery to hepatocytes using a novel formulation called Glc-PEG-PEI, composed of galactose, poly(ethylene glycol) (PEG), and (PEI). Their study suggests that Glc-PEG-PEI has superior transfection efficiency compared to conventional PEI carriers. This finding highlights Glc-PEG-PEI as a promising candidate for liver-targeted gene delivery [63]. He et al. synthesized poly(5-methyl-5-allyloxycarbonyl-trimethylene carbonate) using immobilized porcine pancreas lipase [64]. Researchers enhanced PMAC by incorporating epoxide groups and PEI to create PMAC-g-PEI, which exhibited improved transfection efficiency and reduced cytotoxicity in 293T cells compared to PEI alone. Subsequent modification with DTC produced P(MAC-co-DTC)-g-PEI, a promising gene delivery vector [65]. Scheme 1 below depicts the synthesis process for P(MAC-coDTC)-g-PEI. The investigation of several facets of PEI utilization is presently in progress.

##### PEI as a Co-Delivery System for Drugs

PEI utilized as a drug co-delivery system can be found in linear and hyperbranched (or branched) forms, with its properties and applications varying significantly depending on the structure. PEI is known for its effectiveness and cost-efficiency and is widely used in various applications, particularly as a nucleic acid transfection agent. Studies have shown its potential as a co-delivery system, such as when combined with amine-functionalized biochar or PEI-functionalized magnetic Fe_3_O_4_ systems. These combinations enhance drug release and functionality [66,67]. Additionally, PEI and its derivatives, like N-Ac-L-Leu-PEI, show promise for gene delivery, particularly for CpG oligo-deoxy nucleotides. These oligo-deoxy nucleotides are crucial for inflammation resistance, bone resorption, and modulation of cell apoptosis [68]. Yoshitomi et al. observed an enhanced accumulation of astaxanthin in Haematococcus pluvialis cells upon PEI addition, attributed to increased reactive oxygen species production and oxidative stress [69]. PEI versatility extends to serving as a co-delivery system in various research areas, including anticancer drug delivery, cancer gene therapy, and drug adsorption enhancement.

Docetaxel stands as a cornerstone in chemotherapy, particularly for resistant prostate cancer [70], yet formidable drug resistance impedes its clinical efficacy. Combining gene therapy with chemotherapy, a durable strategy, is proposed due to DTX’s varied resistance mechanisms, although the differing properties of DNA and chemotherapy drugs pose challenges in carrier delivery [71]. TAT peptide, derived from HIV-1, enhances gene vector uptake and transfection efficiency, offering a potential solution. Dong et al. engineered TAT-PEG-PEI-OA, a sophisticated PEI-based carrier integrating TAT peptide, oleic acid, and PEG, facilitating the concurrent delivery of pDNA and DTX. Characterized by a particle size of 270 nm and a zeta potential of 22 mV, the complex exhibited notable cytotoxicity against tumor cells, with 1.5 times higher transfection efficiency than control groups after 24 h, suggesting potential for sustained drug delivery within tumor tissues [72].

#### 3.1.2. Hyperbranched Polymers

Hyperbranched polymers are synthesized through a one-pot polymerization process involving multifunctional monomers, which leads to a highly branched structure.

##### Hyperbranched PEI

**Figure 7:**
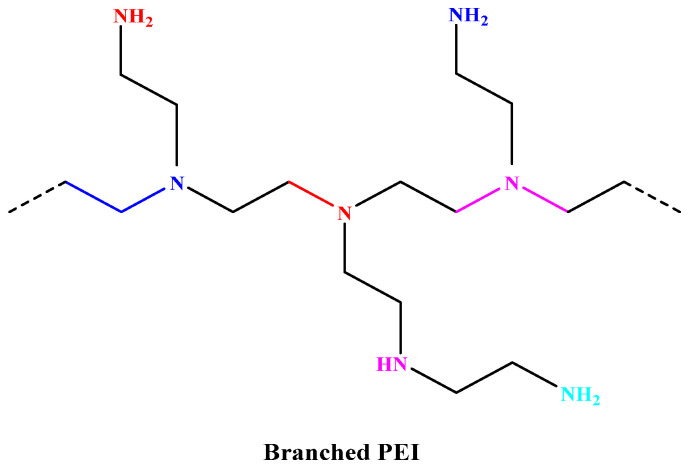
Structure of branched PEI.

Branched polyethyleneimine is a hyperbranched polymer synthesized via the acid-catalyzed ring-opening polymerization of aziridine monomers. Each branch contains 3–35 nitrogen atoms, forming a spherical internal structure that can encapsulate nanoparticles, drug molecules, and other small molecules [73]. PEI is a synthetic branched polymer of ethylene imine monomers [74]. Branched PEI has a unique structure containing numerous primary, secondary, and tertiary amines [75]. The synthetic structure of branched H(NHCH_2_CH_2_)_n_NH_2_) is shown below in Figure 7.

**Scheme 2:**
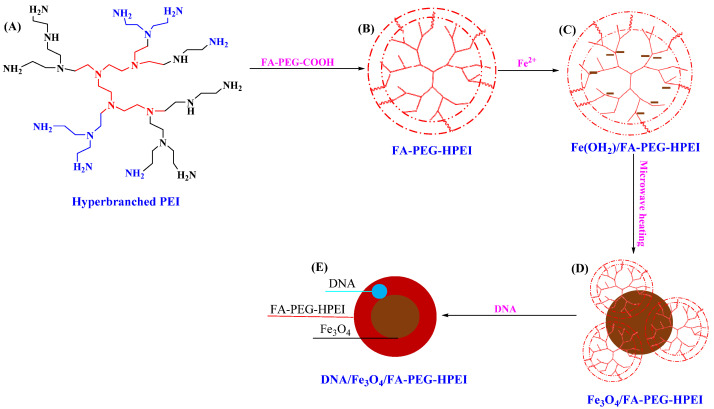
Illustration of the synthesis of folate-targeted and PEG-modified HPEI (FA-PEG-HPEI) (**A**,**B**), preparation of Fe_3_O_4_ nanocarriers (NCs) within FA-PEG-HPEI (**B**–**D**), and their subsequent magnetofection study (**E**) [78].

Chen et al. created a high-performance PEI-graphene oxide (GO) composite gene vector by reacting carboxyl groups with amino groups. The resulting branched PEI-GO showed reduced toxicity and increased transfection efficiency compared to 25 kDa PEI [76]. Cook et al. synthesized a hyperbranched poly(ethylene imine-co-oxazoline) via thiol-yne reaction and acid hydrolysis. While it showed reduced cytotoxicity, its transfection efficiency was slightly lower than the standard, indicating a need for further modifications to enhance performance and suitability for various applications [77]. Hyperbranched poly(ethylene imine) (HPEI) is a leading non-viral gene vector but faces challenges with dose-dependent cytotoxicity and lack of targeting. PEG was grafted onto HPEI to address these issues, lower cytotoxicity, and improve gene transfection. A folate moiety was also attached to PEG for targeted delivery to cancer cells. This led to the development of folate-targeted, PEG-modified HPEI (FA-PEG-HPEI), as shown below in Scheme 2A,B [78].

FA-PEG-HPEI, with its hydrophilic FA-PEG chains and a hyperbranched HPEI core rich in amines, chelates ferrous ions and provides alkali for Fe_3_O_4_ nanocrystal synthesis. In a nitrogen atmosphere, ferrous ions bind to the amines and react with the alkali, forming ferrous hydroxide/FA-PEG-HPEI. Through microwave digestion, heating, aging, and dialysis, Fe_3_O_4_ NCs stabilized by FA-PEG-HPEI are produced, forming Fe_3_O_4_/FA-PEG-HPEI nanocomposites, as shown in Scheme 2B–D. Exposure to air during synthesis and transfer converts some ferrous hydroxide to ferric hydroxide, which forms Fe_3_O_4_ NCs upon microwave heating. These nanocomposites are magnetic, folic acid-targeted gene vectors, as illustrated above in Scheme 2E [78].

##### Poly(*β*-amino ester) (PβAE)

**Figure 8:**
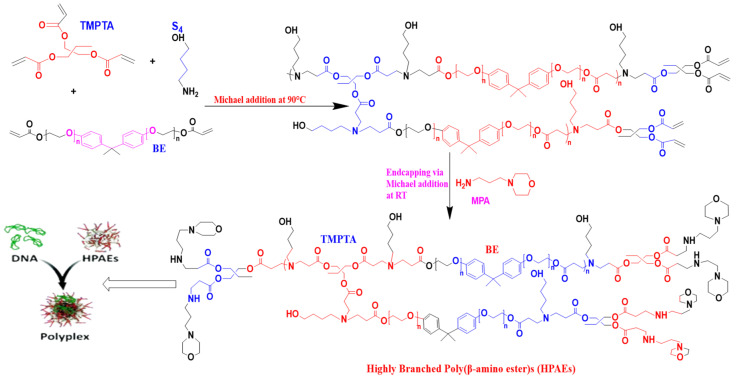
Synthesis of highly branched P*β*AE [83].

P*β*AE is a cationic polymer synthesized by reacting acrylates with amines via Michael addition reactions [79]. P*β*AE is biodegradable, biocompatible, and pH-responsive, making it an attractive option for nucleic acid delivery applications [80]. The polymer forms backbones with ester bonds easily degraded by hydrolysis under physiological conditions. Upon hydrolysis, the polymer breaks down into small molecules, such as bis(*β*-amino acids) and diols, which are considered harmless to mammalian cells [81]. Highly branched P*β*AEs are synthesized using triacrylates and an amino moiety with two reactive sites, as illustrated below in Figure 8. Moreover, highly branched P*β*AEs have been used to deliver minicircle DNA to treat neurological diseases [82].

P*β*AEs are excellent transfection agents, and they have been used in many gene delivery applications. For example, P*β*AEs have demonstrated great transfection efficiency with little cytotoxicity when employed to introduce plasmid DNA into cells for gene therapy. P*β*AEs formed stable polyplexes with nucleic acids, which allowed them to transfer genes more effectively than traditional cationic polymers like PEI, according to one research. Utilizing P*β*AEs to transfect primary cells was another application that demonstrated the potential of these particles for therapeutic gene transfer. P*β*AEs have also been investigated for the delivery of RNA molecules, such as messenger RNAs (mRNAs) and small interfering RNAs (siRNAs), with encouraging outcomes for research on protein expression and gene silencing [84].

#### 3.1.3. Dendritic Polymers

Dendrimers are highly branched polymers with well-organized 3D morphology that possess perfect symmetry or near-perfect symmetry to deliver drugs, including biological-based drugs, such as genes. Dendrimers are 3D structures that could be described as resembling snowflakes. They feature a central core with branches extending outward in a perfectly ordered, repetitive pattern, creating a precise and uniform architecture. They are produced by repeatedly assembling many layers around a central molecule through covalent conjugation [85].

##### Dendrimers

**Figure 9:**
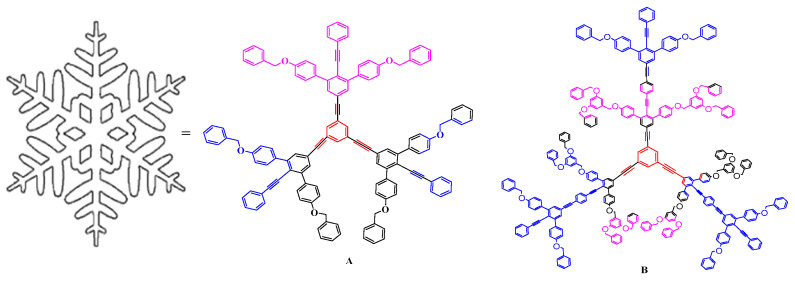
The highly symmetrical physical architecture of the dendrimer mimics a snowflake—that is capable of featuring a unique electron-carrying path within an encapsulated π-conjugated system [87].

Dendrimers are a revolutionary tool for non-viral gene delivery, offering a safe and efficient alternative to traditional viral methods. These nanoscale polymers have a unique tree-like branching structure, allowing precise control over their size, shape, and surface functionality. The highly branched and regularly repeating building blocks of dendrimers effectively isolate the core, creating a specific microenvironment. This isolation of an encapsulated redox-active core has gained attention as a model for biological systems and charge injection into nanoscale devices [86]. As shown in Figure 9 below, due to the highly symmetrical physical architecture of the dendrimer, it mimics a snowflake that is capable of featuring a unique electron-carrying path within an encapsulated π-conjugated system. Unlike previously reported types, this dendrimer cannot be synthesized using standard divergent or convergent methods. Site-selective synthesis is crucial, as the encapsulating part’s size varies with the π-conjugation sites, and is larger in the inner and outer regions.

A successful synthesis method utilizing a dialkyltriazeno group as a key protecting group demonstrates the formation of snowflake-like dendrimers A and B. These dendrimers contain linear oligo(phenylene ethynylene) molecular wires within branched poly(benzyl ether)s, as illustrated above in Figure 9 [87,88]. By complexing with genetic material, snowflake dendrimers protect it from degradation and facilitate cellular uptake through endocytosis. Once inside, the dendrimer releases the genetic payload, enabling the expression of the intended gene. With their high degree of branching and biocompatibility, snowflake dendrimers have shown great promise in delivering large genetic payloads with high efficiency and specificity. Snowflake dendrimers are poised to make a significant impact in the field of gene therapy, potentially treating a wide range of genetic diseases. Dendrimers have several uses in medication delivery and biomedicine. Poly(amidoamine) (PAMAM) dendrimers, for example, are employed for targeted drug administration as well as nanoparticles in imaging. PAMAM dendrimers also promote transdermal medication administration by enhancing drug penetration through the skin [89].

##### PAMAM–Polyamidoamine

**Figure 10:**
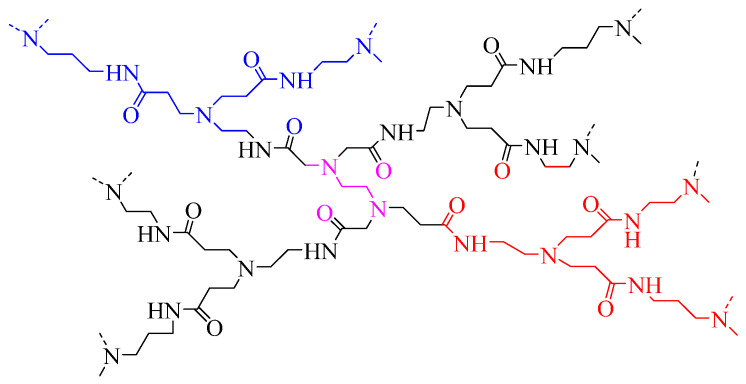
Dendritic structure of PAMAM [91].

The branched, tree-like structure of hyperbranched and dendritic polymers, such as PAMAM dendrimers, is produced via repeated chemical synthesis processes. These polymers have a high concentration of amine groups on their surfaces, enabling effective condensation and DNA binding. Compared to linear polymers, hyperbranched and dendritic polymers exhibit higher transfection efficiencies. PAMAM is the dendrimer most frequently used for non-viral gene delivery, as shown in Figure 10 below. “PAMAM generation” describes the number of consecutive steps synthesizing PAMAM dendrimers. PAMAM’s molecular weight, surface charge, and cytotoxicity increase with each increasing generation [90].

PAMAM has substantial cytotoxicity and quick bloodstream clearance but has superior flexibility, lesser immunogenicity, and higher transfection efficiency compared to other polymeric carriers [92]. The positive charge on PAMAM’s surface induced by its terminal amino groups causes cytotoxicity [93]. Due to their high reactivity, chemical changes are typically made to the terminal primary amine groups that exist on the surface of the dendrimers to reduce their toxicity. Various chemical modifications of PAMAM dendrimers serve to address specific challenges in biomedical applications as shown in Table 2 below. These modifications include enhancing hydrophobicity/lipophilicity balance by incorporating alkyl carboxyl, PEG, and cholesteryl chloroformate [93]. Biodegradability is improved by introducing GLFG oligopeptide and a thioketal core [94,95]. PEG attachment via a disulfide bond and carboxy betaine acrylamide incorporation prolongs circulation and reduces clearance. This modification enhances the vector’s stability and bioavailability [96,97].

Additionally, cell binding and nucleic acid binding improvements are achieved by utilizing a gold nanoparticle core and PAMAM-coated liposomes [97,98]. Modifications such as ASSLNIA oligopeptide and the inclusion of monosaccharides like glucose and mannose are implemented to target specific tissues [99,100]. Pishavar et al. varied PEGylation, alkyl carboxylation, and cholesteryl chloroformate addition ratios to enhance the complexes’ hydrophobicity and lipophilicity balance [93].

**Figure 11:**
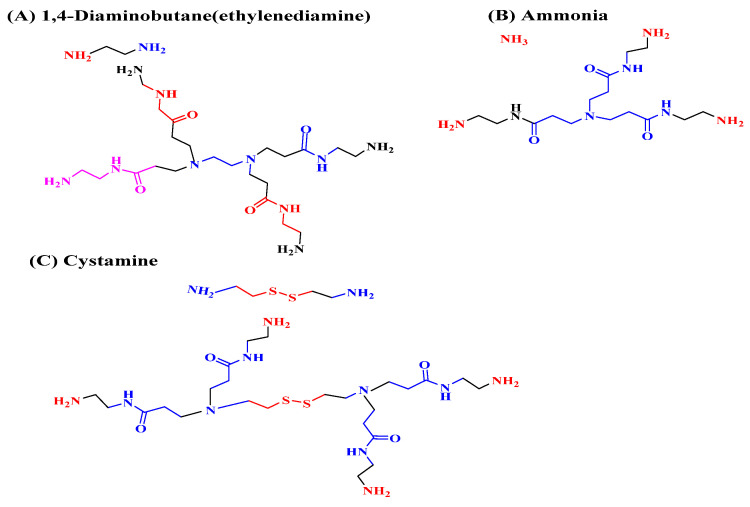
Standard cores—(**A**) 1,4-diaminobutane(ethylenediamine), (**B**) ammonia, (**C**) cystamine and G0 PAMAM derivatives of each core [90].

PAMAM is the most extensively studied and well-characterized dendrimer class, being the first to be synthesized and commercialized [101]. Dendrimer synthesis can be conducted in various ways, the most widely utilized being convergent and divergent methods. Some innovative techniques have been developed in the past few years, including joint convergent–divergent, click synthesis, hyper cores, branching monomers, double exponential, and Lego chemistry [102]. Primary amine-containing linear chain molecules can make up the PAMAM dendrimer core. As seen below in Figure 11, the most common dendrimer core molecules are ethylenediamine, ammonia, and cystamine, offering multiple branching points for dendrimer growth.

Dendrimer generations are produced through a repetitive two-step process involving Michael addition reactions and alkyl acrylate, yielding terminal ester groups [103,104]. PAMAM dendrimer generations are produced through ester amidation with ethylenediamine, resulting in exponential increases in molecular weight, atoms, and terminal amine groups, with a 10 Å radius increase per generation. Dendrimer growth patterns shift from linear to globular shapes with increasing generations, creating cavities suitable for encapsulating and adsorbing biomolecules, making them ideal for biomedical applications [104]. PAMAM’s dendritic properties, characterized by tree-like architecture and exponential growth, distinguish it from other polymers, enabling enhanced drug delivery, controlled release, and encapsulation of imaging agents [105]. Mastorakos et al. show that amine-functionalized hydroxyl-terminated (PAMAM) dendrimers efficiently compress plasmids. They also showed that triamcinolone acetonide improves the dendrimer-gene complex’s nucleus localization, significantly increasing cell intake and transfection efficiency [106]. At the same time, high-generation PAMAM dendrimers are highly effective for gene transfection, but their cytotoxicity and high cost limit their broad applicability. To address this, conjugation with reactive oxygen species (ROS)-responsive poly(propylene sulfide) (PPS) has led to the development of low-cost amphiphilic PAMAM dendrimers that exhibit high transfection efficiency, low cytotoxicity, and efficient DNA compression and release capabilities [107].

**Figure 12:**
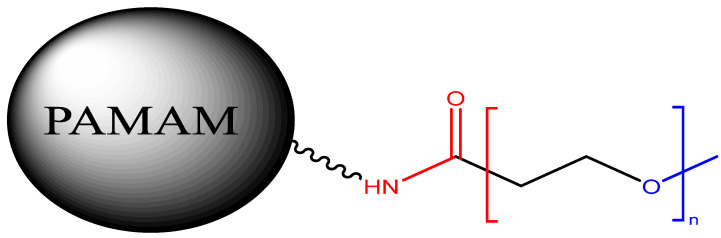
PEGylation is one strategy for decreasing PAMAM toxicity [110].

The primary concerns regarding PAMAM dendrimers are toxicity and safety, as their cytotoxicity—predominantly higher in cationic derivatives compared to neutral or negatively charged ones—depends on concentration, charge, and generation, with toxicity increasing with higher generation and concentration [108,109]. Several studies, including those by Wang et al., have demonstrated a decrease in PAMAM toxicity through PEGylation [110], as shown below in Figure 12. 

Najlah et al. functionalized PAMAM-G0 and PAMAM-G3 with diethylene glycol and lauroyl chains to reduce naproxen’s cytotoxicity and enhance its pharmacokinetic profile, introducing a novel surface modification approach [111]. This study showed improved transport of naproxen conjugates across Caco-2 cells with reduced cytotoxicity, using DEG, lauroyl, and pyrrolidone derivatives of PAMAM-G0 and PAMAM-G3, enhancing their potential for drug delivery applications [112,113], and half-generation anionic PAMAM has shown very low cytotoxicity, lytic, and hemolytic properties across a wide concentration range, as well as no in vivo toxicity, indicating its potential for future biomedical applications.

#### 3.1.4. Comb Polymers

**Figure 13:**
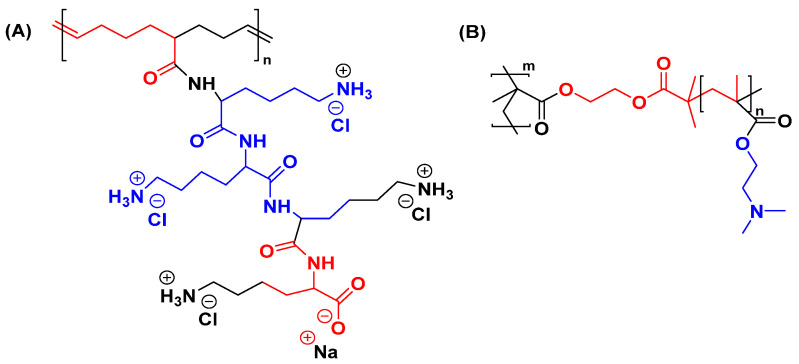
Typical chemical structures of comb polymers comprised of hydrophobic backbone with oligolysine pendent groups [114].

Comb polymers are polymers characterized by a main backbone (the shaft) with a dangling functional group that is part of the repeat unit (the teeth). Comb polymers have emerged as a promising tool in gene delivery, offering high transfection efficiency and low cytotoxicity. These polymers comprise a hydrophobic backbone with oligolysine pendent groups, forming stable polyplexes with DNA [114]. Studies have shown that comb polymers outperform many commercial reagents in transfection efficiency while maintaining high cell viability, as shown in Figure 13A. The hydrophobic backbone constrains interactions with DNA, reducing binding free energy and enhancing transfection efficiency. Further modifications, such as incorporating zwitterionic components, aim to reduce cytotoxicity and enhance colloidal stability.

**Figure 14:**
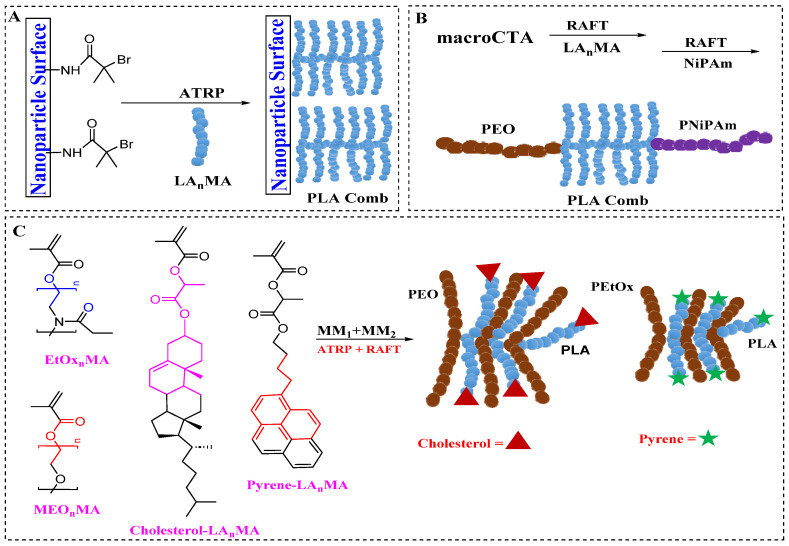
Schematic of comb polymer synthesis with PLA side chains via the macromonomer approach. (**A**) Surface-initiated polymerization of the macromonomer. (**B**) Use of a macroCTA for macromonomer polymerization, followed by NiPAm chain extension, forming a palm tree-like structure. (**C**) Copolymerization of different macromonomers to produce heterografted comb polymers [116].

Olden et al. showed that poly(2-dimethylaminoethyl methacrylate) (pDMAEMA)-grafted comb polymers have demonstrated high transfection efficiency and cell viability in human T-cell transfection, as shown above in Figure 13B [115]. The success of comb polymers in gene delivery has led to the exploration of their combination with physical delivery techniques, such as sonoporation, for enhanced in vivo delivery. Overall, comb polymers offer a promising approach to gene delivery, with their unique structure and properties enabling efficient and safe transfection of cells [114]. Polylactic Acid (PLA) is an aliphatic polyester formed via the ring-opening polymerization of lactide monomers. The homopolymerization of methacrylate-functional PLA macromonomers via RDRP creates PLA comb polymers with a polymethacrylate backbone, as shown in Figure 14 below. Synthetic methods include grafting from surfaces, chain extension with small monomers, and copolymerization with other macromonomers. Research focuses on comparing these dense polymers with their linear counterparts [116].

Wu et al. synthesized a triblock copolymer (PEO-b-PHEMA-g-PLA-b-PNiPAm; Ð = 1.35) via RAFT polymerization of LA5MA using a PEO macro-CTA, followed by NiPAm chain extension [117]. PLA macromonomers with a methacrylate ω-end functionality were synthesized using functional initiators during ROP. Following the post-radical polymerization of the comb polymer backbone, the α-end functionality decorates the side chain ends, as shown in Figure 14 [116]. Poly(L-lysine)-grafted-poly(ethylene glycol) (PLL-g-PEG) is an example of a comb polymer.

##### Poly(L-lysine)-grafted-poly(ethylene glycol) (PLL-g-PEG)

**Figure 15:**
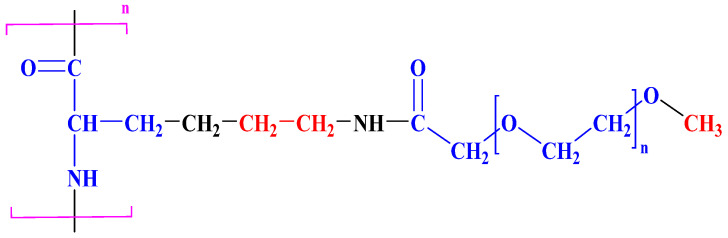
Chemical structure of PLL-grafted-PEG.

Nanomaterials are widely used to deliver treatments, including anticancer medicines, peptides, proteins, and nucleic acids [118,119]. One critical aspect of nanoparticle (NP) delivery systems is the material utilized for NP synthesis. The cationic grafted copolymer PLL-g-PEG, where the hydrophilic PEG component is extensively used to reduce clearance and enhance the biocompatibility of biomaterials, NPs, and pharmaceuticals [120], as shown below in Figure 15. PEGylation, or PEG coating, effectively prevents liver and kidney clearance of molecules and is commercially employed to improve the circulation characteristics of several proteins, including asparaginase and interferon [121,122].

PEG imparts beneficial properties by reducing serum protein adsorption onto NP surfaces, preventing protein adsorption, and decreasing NP cellular uptake to about 10% of that observed with comparable uncoated NPs [123], with its molecular properties—such as the molecular weight and extent of copolymer grafting—also influencing this adsorption. Rausch et al. discovered that the 10–20% PEGylation of PLL effectively prevents the formation of large aggregates with serum proteins [124]. At the same time, Gref et al. determined that 5 kDa PEG is optimal for avoiding plasma protein adsorption on the surface of PLA NPs [125]. PLL-g-PEG’s ϵ-amino groups, which are cationic at physiological conditions, allow the polymer to electrostatically self-assemble with negatively charged molecules, generating nanoparticles called polyion complexes [126]. Synthetic gene delivery applications have used PLL and other cationic polymers to form complexes with nucleic acids, leveraging specific properties to enhance delivery efficiency [127,128,129].

#### 3.1.5. Brush Polymers

The brush-like polymer is a unique branching structural polymer created by grafting short-side chain molecules onto the backbone chain [130]. Macromonomers are macromolecules with one or more functional groups that allow them to act as monomers for polymerization [131]. The three methods of “grafting-through” (the brush-like polymer is polymerized via macromonomers polymerization), “grafting-to” (the brush-like polymer is prepared by grafting the side chain integrally to a backbone), and “grafting-from” (the brush-like polymer is constructed through monomers’ polymerization from a backbone) were generally used in the synthesis of the brush-like macromolecules [132,133]. Well-defined brush polymers with a high grafting density can be achieved through the polymerization of macromonomers [133]. 

Li et al. discovered that brush cationic polymers, namely poly(oligoethylene glycol) methacrylate-cationic hyperbranched polymers, exhibit enhanced biocompatibility, lower steric hindrance, and improved siRNA binding and gene silencing efficacy, making them promising for treating heterotopic ossification [134]. Burdynska et al. used the grafting-from method to generate bottle-brush polymers with shorter and longer grafted chains or a limited molecular weight variation [135]. Nese et al. created a pH-responsive fluorescent brush polymer by grafting fluorescein in O-methacrylate onto the brush polymer’s side chain using ATRP. This polymer showed prominent fluorescence intensity characteristics in essential situations and did not fluorescent in neutral or acidic environments [136]. Chen et al. developed a unique brush-on-brush architectural polymer by integrating ATRP, ring-opening polymerization (ROP), and a click chemical reaction, with poly(oligo(ethylene glycol) acrylate) acting as the brush. Brush-on-brush architectural polymers show different chain lengths to mitigate the repulsion between them, which can be achieved by increasing the density and dimensionality of the chains on each side [137].

**Scheme 3:**
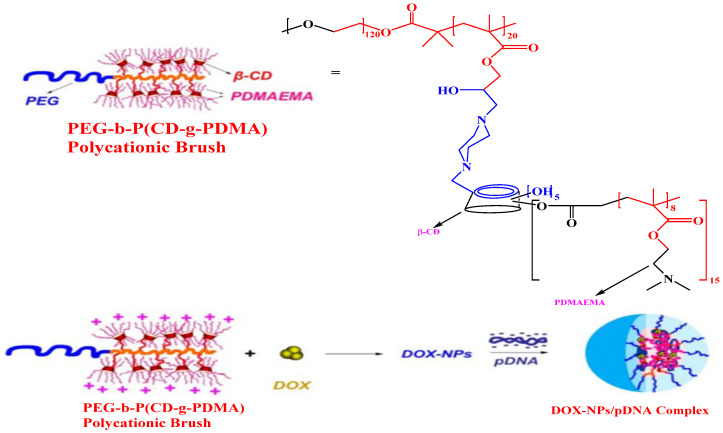
Chemical structure of the poly-cationic brush, preparation of doxorubicin-loaded nanoparticles (DOX-NPs), and multifunctional DOX-NPs/pDNA complexes [139].

Polymer brushes are materials with ultra-dense chains extending from a surface where the distance between neighboring chains is less than twice the gyration radius of a free polymer chain [138]. A well-defined coil-comb poly-cationic brush with “CD-containing cationic star polymers” as side chains were synthesized via atom transfer radical polymerization, as illustration below in Scheme 3.

Combining brush-like architecture with CD-based star polycations, this brush structure showed superior gene transfection efficiency in COS-7 and 293T cells compared to individual star polycations or PEI25K. We used it to develop a multifunctional carrier for the co-delivery of doxorubicin (DOX) and a p53-encoding plasmid [139]. Wang et al. suggested that a PEG-based brush polymer containing disulfide links may facilitate the particular release of siRNA to cancer cells [140]. Also, cellular intake, in vitro transfected efficiency, and post-transfection survival of cells all indicated that the brush-like shape improved nuclease stability, cell uptake, and siRNA distribution. In particular, its blood removal half-life was raised 19 times. The PEG-bottle brush polymers’ anti-tumor properties and safety in vivo supported their use as an efficient long-circulating carrier for siRNA silencing therapies. Blum et al. found that the amount of cell penetration peptide arms significantly impacts the gene editing efficacy of brush polymers, emphasizing that functionalized arms efficiently promote the editing of genes and protein delivery [141].

Additionally, Ahern et al. showed that the hydrophobicity and charge density of the arms affected the cytotoxicity and efficacy of polymer-mediated transfection [142]. Nie et al. prepared organic base brush polymers for gene transfer by grafting polymethacrylic acid onto the side chain of heparin, owing to the natural material’s high biocompatibility [143]. Therefore, various side chain changes can discuss several features of brush polymers to fulfill the different needs of gene transport, such as toxicity to cells, mitochondrial escape, and nuclear localization.

#### 3.1.6. Star Polymers

**Figure 16:**
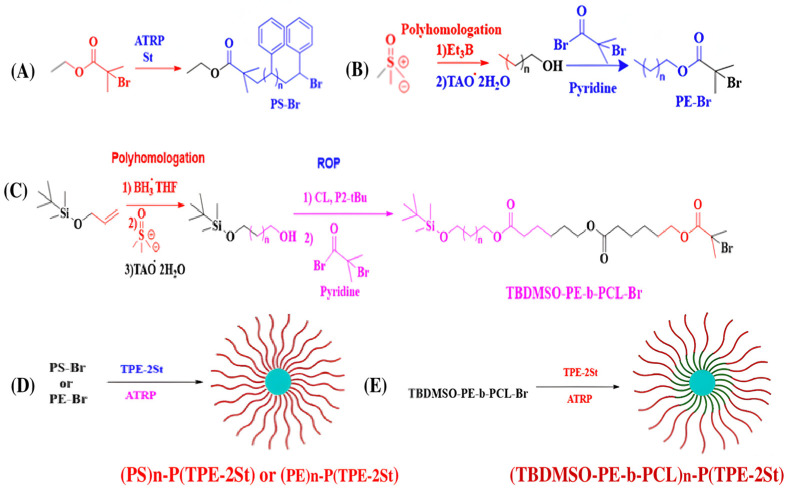
Arm-first synthesis of a star-shaped polymer that employs the use of double styrene-functionalized tetraphenylethene displaying aggregation-induced emission (AIE) traits as the core and polystyrene, polyethylene, or polyethylene-b-polycaprolactone as the arms (**A**–**E**) [151].

Star polymers were frequently employed for gene transfer due to their precise structure, flexibility of modification, and increased transfection effectiveness [144,145]. Star polymers are another example of branched macromolecules with linear polymer chains covalently attached to a central core, so the final structure has one branched point per macromolecule. Recently, their simplicity of synthesis, potential for high molar mass, versatility, and distinct characteristics have sparked great interest for use in gene delivery applications. Many star polymers with polycationic arms have been produced for gene transfection. However, their efficacy is frequently hampered by toxicity [146,147]. Studies have revealed that the chemical structure and molar mass of these star polycations substantially impact their toxicity to treated cells [148,149]. Ding et al. created star polymers by blocking polymer arms using electron transfer ATRP. Scientists first produced poly(butyl acrylate-tert-butyl acrylate) using a linear block to make a multi-arm star block polymer. Then, they crosslinked the end groups of the linear molecules with divinylbenzene [150]. To create a multi-arm star polymer using an arm-first method that produced a 70% yield in two steps, Zhang et al. used double styrene-functionalized tetraphenylethene displaying aggregation-induced emission (AIE) traits as the core and polystyrene, polyethylene, or polyethylene-b-polycaprolactone as the arms, as shown below in Figure 16 [151]. Yoshizaki et al. used the arm-first approach to rapidly and quantitatively create different star poly(p-methoxystyrene) using active cationic polymerization [152].

**Figure 17:**
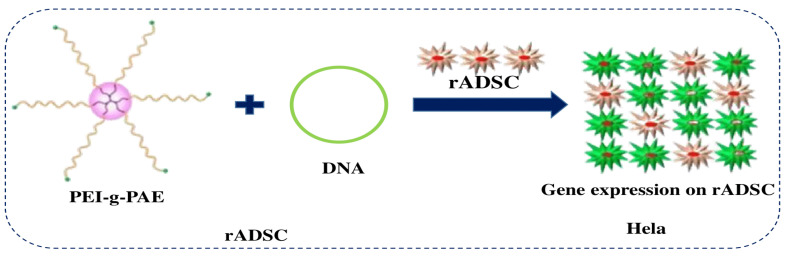
Synthesis of star PEI-g-PAE via the grafting-onto approach for gene delivery [155].

Cho et al. demonstrated in vitro that PEG-armed star polymers could efficiently transfer DNA and siRNA to S2 cells [153]. This study explores the potential of star- and sun-shaped polymers with hyperbranched cores for gene delivery, providing a valuable reference for future research. A polypeptide-PEG miktoarm star copolymer showed high cellular uptake and transfection efficiency in A549 cells, with moderate cytotoxicity, as shown below in Figure 17 [154].

The miktoarm star copolymer achieved 68% luciferase gene silencing efficiency at a 150 nM siRNA dose and enabled intracellular transport pathway visualization, allowing for combined gene delivery and bioimaging. A star polymer with P(DMAEMA-co-OEGMA-OH) arms efficiently delivered DNA and mRNA, providing insights for creating versatile genetic material delivery systems [148]. Building on the advantages of PEI and PAE in gene transfer, Huang et al. utilized a grafting-to technique to develop a distinctive star-shaped PAE polymer, as shown above in Figure 17. This polymer consists of low molecular weight PEI at the core, with low molecular weight LPAE forming the arms. This modified star-shaped PAE displayed improved gene transfection efficacy and reduced cytotoxicity in ADSCs, outperforming individual PEI and LPAE by 264-fold and 14,781-fold [155]. Wang et al. proposed using hyperbranched-star PEI-g-PEG as a polycationic gene carrier to create non-viral vectors for potential retinoblastoma gene therapy. The PEI-g-PEG effectively condenses genes, and by optimizing the composition proportions, cationic nanoscale complexes with PEG shells were obtained, ensuring efficient uptake and low toxicity [156].

#### 3.1.7. Hydrogels

**Figure 18:**
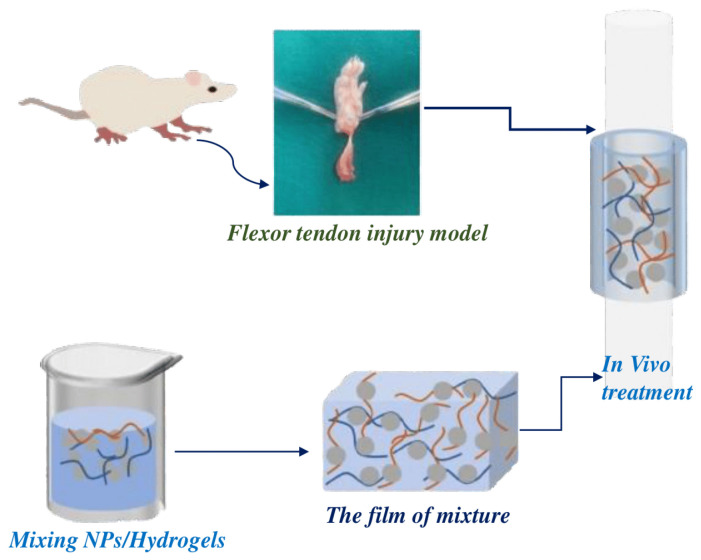
Illustrates hydrogel use in vivo. This mesoporous polymer can encapsulate nanoparticles with target genes, forming a film at room temperature. The hydrogel film can treat flexor tendon damage [161].

Hydrogel, a three-dimensional (3D) hydrophilic polymer network primarily composed of water, is analogous to the physical microenvironment [157]. Hydrogels, which come in a variety of physical forms including slabs, microparticles, nanoparticles, coverings, and films, are frequently utilized in clinical practice and scientific research for applications such as transplantation and regenerative medical testing, cellular immobilization, which involves biological molecules or cell division, and as obstacle materials to regulate physiological adhesions [158]. Hydrogels are excellent delivery vehicles for hydrophilic macromolecules such as DNA due to their high loading ability and encapsulated vectors in the hydrated gel. Their permeability, biocompatibility, and deformability make them ideal for applications in medicine, with natural biological polymer hydrogels, such as chitin, alginate, gelatin, collagen, and hyaluronic acid, demonstrating higher DNA encapsulation efficiency and less DNA damage than synthetic polymers [159]. Hydrogel is a mesoporous polymer network that can hold pharmaceuticals and be customized for target tissues. Hydrogels for tendons or ligaments should have a tensile strength of 10–100 MPa, fracture toughness of 20–30 kJ m^−2^, and a fatigue threshold of 1000 J m^−2^. Additionally, hydrogels can prevent tendon adhesion, demonstrating their versatility in treating tendon issues due to their toughness, strength, and elasticity [160]. In Figure 18 below, one can see how the hydrogel is applied in vivo.

Schulze et al. encapsulated PEI-modified polyplexes and lipopolyplexes in PVA hydrogels, forming NiMDS. These hydrogels offered targeted, long-term nanoparticle release. This was achieved by controlling PVA crosslinkers and molecular weights [162]. Hydrogels manufactured from synthetic polymers, such as modified PEG and amphiphilic block copolymers, provide more control over release characteristics [163,164]. Gelatin hydrogels were utilized to distribute FGF plasmids, improving blood vessel development by delaying gene breakdown for up to 28 days after being injected into hind limb skeletal muscle, resulting in enhanced gene transfer capacity and decreased muscle necrosis for four weeks [165].

Similarly, alginate hydrogels have been used for VEGF plasmid distribution [166], while fibrin hydrogels have encapsulated and distributed HIF-1a plasmids [167]. Kong et al. created a degradable hydrogel by combining different molecular weight alginates to regulate the rate of pDNA release [165]. The polymer segments responsible for ionic crosslinking were designed to be mismatched in size. Using hydrogels to transport PEI/VEGF compounds increased angiogenesis and blood perfusion in mice with ischemic hind limbs [166]. Despite their numerous benefits, hydrogel’s poor mechanical properties limit their use in load-bearing applications. This disadvantage may result from the early dissolving or movement of the gel in the targeted site [158].

#### 3.1.8. Natural Polymers/Modification of Natural Polymers

**Figure 19:**
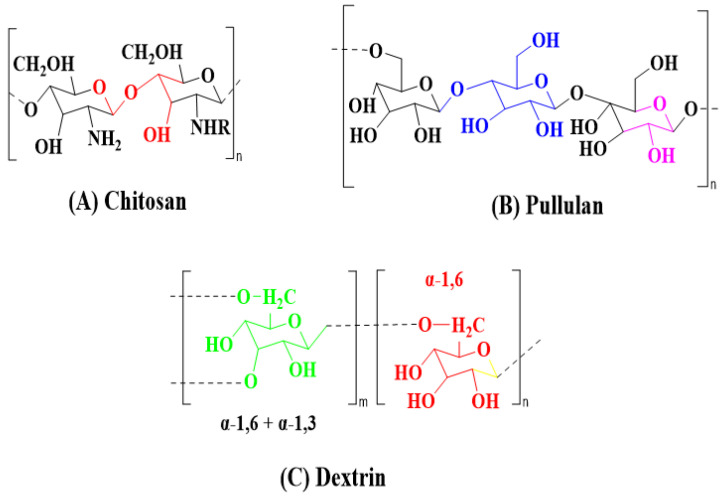
Chemical structures of commonly used natural polymers for gene delivery [173].

Natural polymers have been explored for gene transfer because of their low toxicity and biodegradability [168,169]. Natural polymers typically have changeable active sites, allowing for improved physicochemical properties. Cationic polymers, whether linear or branched, often have amine groups that can be protonated in acidic conditions. The number of proton-able groups varies between cationic polymers, resulting in positive charges dispersed along the leading chains and branches [170]. Natural polymers like chitosan and pullulan are often considered more biodegradable and less toxic, but synthetic polymers can also be engineered for adaptable degradation and high biocompatibility, making both suitable for gene therapy [171,172]. Polysaccharides can adopt a helicoidal structure and are classified as neutral (e.g., dextran), cationic (e.g., chitosan), as illustrated below in Figure 19, or anionic (e.g., hyaluronic acid).

Chitosan, a poly-D-glucosamine from deacetylated chitin, and its derivatives are well-studied gene carriers. Transfection efficiency depends on the deacetylation degree, molecular weight, plasmid concentration, charge ratio (amine to phosphate), serum concentration, pH, and cell type [174]. Chitosan, a modified cationic copolymer of d-glucosamine and N-acetyl glucosamine, is highly biodegradable, biocompatible, mucoadhesive, and antibacterial. Its pH-sensitive nature, with a pKa of around 6.5, facilitates a soluble−insoluble transition between a pH of 6 and 6.5, enhancing its use in tissue engineering, drug delivery, and gene transfection [175]. Chitosan’s mucoadhesive properties enhance the oral and nasal delivery of nucleic acid drugs. Modifications like methylation, PEGylation, and histidinylation improve polyplex stability, endosomal escape, and cellular uptake. PEGylation also increases water solubility and extends half-lives [176]. Nguyen et al. used sodium tripolyphosphate (TPP) to encapsulate miR-33 in polyethylene glycol chitosan polymers (chNPs), creating a vector that targeted mouse macrophages, reducing ABCA1 expression and lowering liposterol outflow in cholesterol metabolism [177]. Zhou et al. modified trimethyl chitosan (TMC) with the REDV peptide and PEG to deliver miR-126 into vascular endothelial cells, enhancing cell proliferation and improving ischemic myocardial necrosis through high transfection efficiency [178].

**Chitosan derivatives:**

Chitosan derivatives with improved solubility for gene delivery have been extensively studied and reviewed [179,180]. Kritchenkov et al. explored hydrophilic and hydrophobic covalent modifications of chitosan, with hydrophilic changes, such as conjugation with PEG, being particularly effective in increasing solubility [181]. Hydrophilic modification of chitosan, mainly through PEG conjugation, significantly enhances its solubility. Jiang et al. found that PEGylation improved solubility, reduced nanoparticle size, and decreased zeta potential without affecting siRNA binding. However, excessive PEGylation reduced cellular uptake and transfection efficiency, making optimization essential. Other hydrophilic modifications for gene delivery include dextran and poly(vinyl pyrrolidone) [179].

Hydrophobic modifications to chitosan, such as conjugation with stearic acid (SA) via the EDC/NHS method, enhance cellular binding, nanoparticle stability, uptake, and DNA dissociation [181,182,183,184]. Chitosan, a biocompatible and biodegradable cationic polysaccharide, forms nanoparticles with negatively charged nucleic acids for intracellular transfection. Its amino groups enhance lysosomal escape via the proton sponge effect. Chitosan is soluble in acidic media but not in neutral or alkaline environments, limiting its gene transfection use. Increasing solubility through low molecular weight chitosan or PEGylation expands its application potential.

**Pullulan:**

Pullulan (PULL), a water-soluble polysaccharide from Aureobasidium pullulans, consists of maltotriose and maltotetraose units linked by α-d-(1 → 6) bonds, forming α-(1 → 4) linked trimeric repeating units, and is ideal for drug delivery [185,186]. PULL is widely used commercially in flocculants, blood plasma expanders, food additives, adhesives, and dielectric materials. The FDA deems it “generally recognized as safe” for various uses, including as an excipient in pharmaceutical tablets, and it is tested as a cancer vaccine vehicle [187,188]. Due to their non-toxic, non-carcinogenic, and non-mutagenic properties, PULL and its derivatives are extensively studied for medication and gene delivery. Additionally, PULL can be chemically modified by utilizing one or more of its nine hydroxyl groups per repeating unit. These modifications can include hydrophobization, thiolation, PEGylation, or conjugation with cationic substituents. PULL is often used for liver targeting due to its strong affinity for the ASGPR in the liver [189,190,191]. PULL-PEI/siRNA complexes were formed to deliver oligonucleotides to the liver [191] effectively. In another study, PULL was used to create a polyplex with pDNA/siRNA for selective accumulation in folate receptor-overexpressing HeLa cells, resulting in a low-cytotoxicity gene carrier that successfully transmitted gene/siRNA [192]. PULL nanocarriers co-delivered doxorubicin and beclin1 shRNA, enhancing the drug’s anticancer effect [193]. An amphiphilic cationic polymer with deoxycholic acid, PEI, and PULL forms micelles with a PEI shell binding shRNA and a DA core encapsulating anticancer drugs, overcoming chemotherapy resistance [194]. Folate-coated PULL-based copolymer targets tumor cells, inhibiting cancer growth more effectively than DOX or shBeclin1 alone [195]. Additionally, cationic PULL derivatives, modified with quaternary ammonium groups, show promise as miRNA carriers. They form non-cytotoxic polyplexes with human umbilical vein endothelial cells, suggesting their potential as delivery platforms for miRNA therapy [196].

**Pullulan as a carrier for gene delivery:**

Gene therapy applications of pullulan are being explored due to its biocompatibility and non-toxicity, addressing the drawbacks of immunogenic and hazardous viral vectors. Pullulan derivatives with metal chelating residues mixed with plasmid DNA in Zn^2+^ solutions create conjugates that target the liver. Chemically modified pullulan derivatives, such as pullulan-Ti, pullulan-DTPA, and pullulan-Sm, show significant enhancement in liver-specific gene expression when intravenously injected, outperforming free plasmid DNA in gene delivery [187]. The pullulan-DTPA–plasmid DNA conjugate significantly enhanced liver-specific gene expression, lasting over 12 days, and was higher than the mixture form. Fluorescent microscopy confirmed liver localization and the pre-injection of arabinogalactan and galactosylated albumin suppressed gene expression, indicating hepatocyte transfection and the promise of Zn^2+^ coordinated pullulan for targeted gene delivery [197].

**Dextran:**

Dextran, a benign and nonimmunogenic polysaccharide, is frequently utilized for gene transfection and medication delivery due to its high water solubility and low toxicity. The chain is composed of α-d-glucose units joined by α-(1 → 6) glycosidic bonds, with varying α-(1 → 4) or α-(1 → 3) links at the beginning [198]. Dextran, produced by gram-positive bacteria like *Leuconostoc* and *Streptococcus* using sucrose, can be chemically modified via etherification, esterification, amidation, and oxidation to enhance its transport capacities [199]. Dextran, initially neutral, undergoes chemical modifications like diethylaminoethyl-dextran [200] or aminoethyl methacrylate [201] to introduce positively charged groups crucial for electrostatic interactions with genetic material [202]. Using carboxymethyl *β*-dextran and protamine sulfate, a tri-drug carrier, enhances docetaxel efficacy against drug-resistant cancers by combining DTX with chloroquine and ATG5-targeting siRNA. This platform, assembled via hydrophobic and electrostatic interactions, showed potent anticancer effects in vitro on MDA-MB-231 cells and in vivo in a mouse xenograft model, effectively suppressing tumor growth and ensuring biosafety for treating triple-negative breast cancer [203]. A pH-sensitive biocompatible dextran nanocarrier for prostate cancer gene delivery targets PSMA via urea-conjugated ligands. With a 40 kDa dextran backbone and acetal-linked amine groups, it undergoes pH-triggered cleavage in acidic endosomes to release siRNA, effectively downregulating the PD-L1 and CD46 genes crucial for immune evasion [204,205]. Dextran and chitosan-based nanoparticles for miRNA delivery were developed, utilizing a redox-responsive polyelectrolyte complex of thiolated dextran and chitosan with miR-145 [206], decorated with anti-nucleolin aptamer AS1411 (apt-PEC) for targeted therapy [207], aimed at increasing the intracellular expression of tumor suppressive miR-145 [208].

**Hyaluronic acid:**

**Figure 20:**
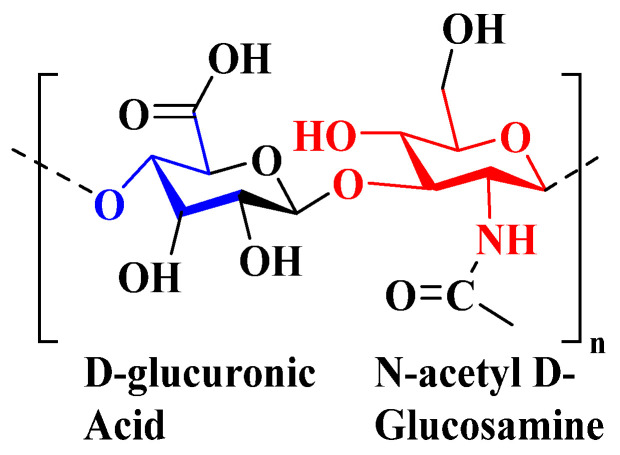
Chemical structure of HA polymers [173].

Hyaluronic acid (HA) is an anionic linear polymer composed of non-sulfated glycosaminoglycan chains with repeating disaccharide units of N-acetyl D-glucosamine and D-glucuronic acid, as shown below in Figure 20, varying in molecular weight from 5 kDa to 20,000 kDa; LMW HA is highly soluble in water, while HA polymers over 200 kDa exhibit high water-holding capacity, crucial for maintaining hydration [209,210,211,212]. HA polymers with molecular weights greater than 1.8 MDa are commercially available, exhibiting remarkable physicochemical qualities like biocompatibility, biodegradability, and non-inflammatory, non-toxic, and non-immunogenic activity [213,214]. Commonly used in medicinal applications such as visco-supplementation, eye surgery, and drug delivery, HA and its derivatives enhance medication delivery across various classes, including antibiotics, antiglaucoma medications, vasodilators, cytokines, and enzymes in both in vitro and in vivo models [215]. It plays a vital role in cell adhesion, growth, and migration [216].

HA’s high binding affinity for CD44 receptors is overexpressed on many tumor cells, which makes it an effective targeting ligand for nanoparticle coating [217]. HA, known for its receptor affinity, enhances cellular uptake synergistically. Its negative charge prolongs therapeutic circulation, shielding against degradation by reactive oxygen species and hyaluronidases in extreme pH conditions. HA nanoparticles (NPs), popular in DDSs, act as carriers alone or with copolymers. They effectively target tumors, delivering genes, xenobiotics, and prodrugs to combat chemo-resistance in cancer therapy [218]. Kim et al. developed an HA-CH-NPs/PLXDC1 siRNA delivery system targeting CD44 receptors on tumor endothelial cells for anti-angiogenic therapy in ovarian cancer [219]. The chitosan particles labeled with HA were loaded with siRNA against PLXDC1, promoting tumor cell migration and invasion. This system effectively targets siRNA to ovarian cancer-associated endothelial cells, protects siRNA from degradation during circulation, induces target gene silencing, and reduces tumor angiogenesis. Fallacara et al. comprehensively reviewed HA, covering its physicochemical properties, biosynthesis, degradation, receptors, industrial production, and cosmetic applications [220]. Knopf-Marques et al. provided an extensive overview of HA and its derivatives in biomaterials, emphasizing hydrogels and coatings for controlled cytokine delivery in implantable environments to mitigate immune responses and enhance tissue healing [221].

## 4. Challenges Facing Polymeric-Based Materials in Non-Viral Gene Delivery

Several challenges facing polymeric-based materials in non-viral gene delivery involve the following:

### 4.1. Stabilizing Genes during Delivery

Ensuring genes remain intact and functional throughout the delivery process is crucial, as degradation can occur due to enzymatic action or physical uncertainty. The intrinsic biochemical properties of the Cas9 protein, plasmid DNA, mRNA, sgRNA, and exogenous donor DNA lead to poor in vivo stability. The Cas9 protein is prone to protease-mediated degradation and hydrolysis, while its positive charge in the blood attracts negatively charged components, causing rapid clearance [222]. Free DNA/RNA is quickly degraded by nucleases in blood and interstitial fluid, requiring protection by delivery vectors. Exogenous plasmid DNA, RNA, and proteins are immunogenic and can trigger immune responses in vivo. The immunogenicity of plasmid DNA can be reduced by removing CpG motifs [223]. Careful chemical modifications have reduced RNA immunogenicity and improved stability. However, the immunogenicity of Cas9 nucleases from *Staphylococcus aureus* and *Streptococcus pyogenes* remains problematic due to frequent human exposure, with pre-existing anti-Cas9 antibodies identified in humans [224,225]. One approach to address this challenge is to engineer bacterial Cas9 nucleases by removing their epitopes or identifying alternative CRISPR nucleases. Despite ongoing efforts to find novel CRISPR nucleases with improved editing efficiency, CRISPR-Cas9 components must be completely encapsulated within delivery vectors to maintain stability and avoid immune responses in vivo [226].

### 4.2. Increasing Capacity for Gene Cargo

Polymeric materials vary in their ability to carry gene cargo, and are influenced by factors like polymer structure, size, and surface properties, limiting the amount of genetic material they can effectively transport. Gene delivery methods vary in cargo capacity; for instance, adeno-associated virus (AAV) vectors typically accommodate up to 4.7 kb of genetic material, expandable to 5.2 kb through modifications. Other vehicles include retroviruses, adenovirus, HSV-1, transposon insertions, episomal vectors, and Sendai virus, each offering distinct advantages such as non-integrating capability. Episomal and HSV-1 amplicon vectors, despite their ability to carry larger payloads, present challenges in purifying supercoiled DNA, necessitating optimization for enhanced delivery efficiency [227,228].

Yu et al. pioneered an episomal reprogramming method for generating feeder-free human induced pluripotent stem cells (iPSCs) using chemically defined media and a small molecule. They successfully derived footprint-free iPSCs from diverse sources, including skin fibroblasts, adipose tissue, and cord blood, showing effectiveness in neonatal and adult fibroblasts. This technique offers promise for clinical applications due to its efficient generation of iPSCs similar to embryonic stem cells (ESCs) [229]. Hou et al. similarly achieved ESC-like iPSCs from mouse somatic cells using a combination of small molecule compounds, showing potential for treating genetic disorders like hemophilia A in a virus-free manner [230]. Stadtfeld et al. demonstrated the efficient generation of mouse iPSCs from fibroblasts and liver cells using non-integrating adenovirus-mediated OSKM expression. These adenoviral iPSCs exhibited typical DNA demethylation patterns, expressed endogenous pluripotency genes, and contributed to various tissues in chimeric mice, including the germline. Their findings support adenoviral reprogramming as a safe alternative without insertional mutagenesis, offering the potential for patient-specific stem cell research and comparative studies with ESCs [231].

Table 3 shows viral vectors and non-viral vectors for gene delivery vectors. Various gene delivery vectors offer distinct cargo capacities and features for genetic manipulation. Retroviruses, capable of integrating genetic material, accommodate 7–10 kb of DNA. Adenoviruses, non-integrating vectors, can carry payloads of approximately 36 kb. AAV vectors, known for safe integration into host genomes, have a cargo capacity of 4.7 kb. HSV-1 amplicon vectors, also non-integrating, boast a high cargo capacity of 150 kb. Tol2 transposon systems, integrating with less frequency, handle payloads of about 10 kb. Episomal vectors, another non-integrating option, range widely in capacity from 172 to 660 kb. Sendai virus vectors, non-integrating as well, possess an enormous cargo capacity of 15,384 kb. These vectors provide researchers with a range of options for delivering genetic material depending on the specific requirements of the experimental or therapeutic application. Cationic polymers like linear PEI have a cargo capacity of approximately 30 kb and are known for their non-integrating nature, making them suitable for transient transfections and effective in plasmid DNA delivery. In contrast, branched PEI has a cargo capacity of around 30 kb but features a branched structure that enhances its transfection efficiency. This structural characteristic makes branched PEI useful for delivering plasmid DNA and RNA.

### 4.3. Targeting Specific Sells

**Figure 21:**
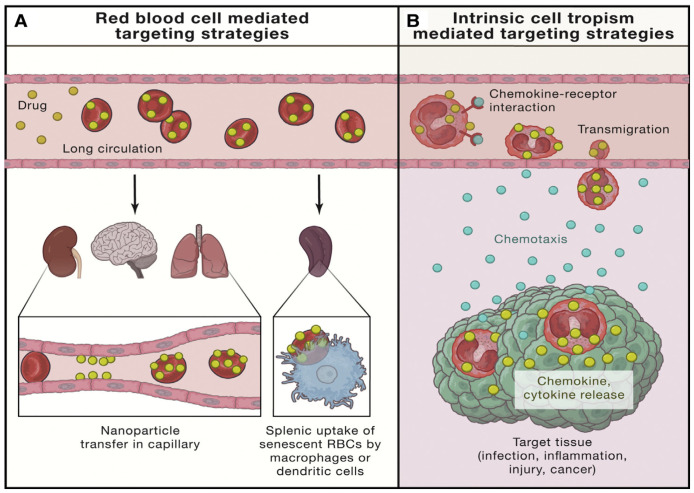
(**A**) RBCs enhance therapeutic circulation and target the spleen through uptake by splenic macrophages or dendritic cells. (**B**) Cells such as leukocytes, stem cells, and platelets migrate to specific tissues, enabling targeted co-delivery of therapeutics [240].

Polymeric carriers can be tailored to target specific cell types through ligands or surface modifications, improving specificity and minimizing off-target effects. DNA and RNA molecules, due to their negative charge and hydrophilicity, struggle to penetrate the hydrophobic, negatively charged cell membrane. While both viral and non-viral vectors offer protection against degradation, they often lack cell type-specific delivery following intravascular administration [240,241]. For systemic CRISPR-Cas9 therapies, cell type-specific delivery is ideal to minimize off-target effects. Nevertheless, local administration can still achieve clinical success, which repairs target cells within specific tissues. Preclinical studies have demonstrated promising results with local CRISPR-Cas9 injections in tissues such as the inner ear, muscle, and brain [242,243,244]. Inspired by natural processes like leukocyte migration, researchers explore natural or engineered cells for site-specific targeting. Although still early, cell-based targeting offers high specificity and versatility. Therapeutics can be attached to or encapsulated within cells, including RBCs, leukocytes, stem cells, platelets, dendritic cells, and bacteria [245,246]. Cell-based targeting leverages masked immune recognition and intrinsic tropism. Cells loaded with therapeutics recognized as self are not quickly cleared. Specific cell types migrate to target sites in response to pathological cues via receptor-ligand interactions, co-delivering therapeutics for site-specific targeting, as seen below in Figure 21.

### 4.4. Combining Gene and Drug Delivery

Polymeric systems can integrate gene delivery with drug delivery, enabling synergistic therapeutic effects or overcoming drug resistance mechanisms in targeted cells. Despite over 2000 nanoformulations in clinical trials, only a few make it to the market due to production procedure variances affecting efficacy and safety profiles. The EMA’s regulatory recognition emphasizes the importance of precise nanomedicine development criteria, highlighting their potential for greater specificity and efficacy than traditional chemotherapeutics [247]. Numerous publications highlight challenges in anticancer nanomedicines and propose strategies to overcome them [248,249,250]. AstraZeneca’s 5R principle—targeting the right patient, tissue, safety, and commercial potential—seeks to tackle translational challenges [251]. Hare et al. stress the importance of using clinically relevant models that reflect tumor biology and patient diversity to improve clinical trial outcomes [252]. Despite promising preclinical results, many nanomedicines fail in clinical trials, often because they are compared to standard chemotherapy due to ethical considerations [253]. Nanoformulations enhance anticancer drug efficacy and reduce toxicity by optimizing pharmacokinetics through the EPR effect [254,255]. These formulations often exhibit prolonged circulation and preferential accumulation in tumor tissues, minimizing the side effects in normal organs [256]. Brand et al. compared the side effects of five nanomedicine groups and found that cytotoxicity is primarily linked to the active substances, not the nanocarrier or formulation [257]. Luan et al. found that anticancer nanomedicines like PEGylated doxorubicin can reduce cardiotoxicity but may increase side effects such as hand–foot syndrome, rash, and pigmentation [258]. Wolfram et al. detailed nanomedicines’ side effects at molecular, cellular, and tissue levels, noting frequent immunological reactions due to spleen and liver accumulation, foreign nature, and ROS [259]. The precise tuning of gene and drug release kinetics in co-delivery systems is challenging, as interactions between co-encapsulated agents can hinder individual release profiles. For instance, poly(lactide-co-glycolide) microspheres demonstrated a significantly reduced release of the co-entrapped epidermal growth factor receptor AODNs and 5-FU compared to individual formulations, despite comparable initial loading efficiencies [260].

### 4.5. Cellular Uptake and Intracellular Trafficking and Localization

Depending on particle size and surface properties, polymeric/gene particles are typically internalized by cells through endocytosis mechanisms, such as clathrin-mediated, caveolae-mediated, or macropinocytosis. Gene cargo supplied by vectors usually exits eukaryotic cells via the cytoplasm. The cargo must reach the nucleus from there to exercise its genetic impact. Depending on the vector type, numerous methods help to transfer genetic material into the nucleus. Viral vectors frequently use nuclear localization signals (NLS) or hijack the host cell’s nuclear import machinery to move via nuclear pore complexes. Non-viral vectors, such as lipid nanoparticles or polymer-based carriers, may use endosomal escape mechanisms or nuclear localization signals to gain nuclear entrance. Once within the nucleus, the payload interacts with the cell’s genetic machinery, causing genes to be expressed or modified as planned [261].

**Figure 22:**
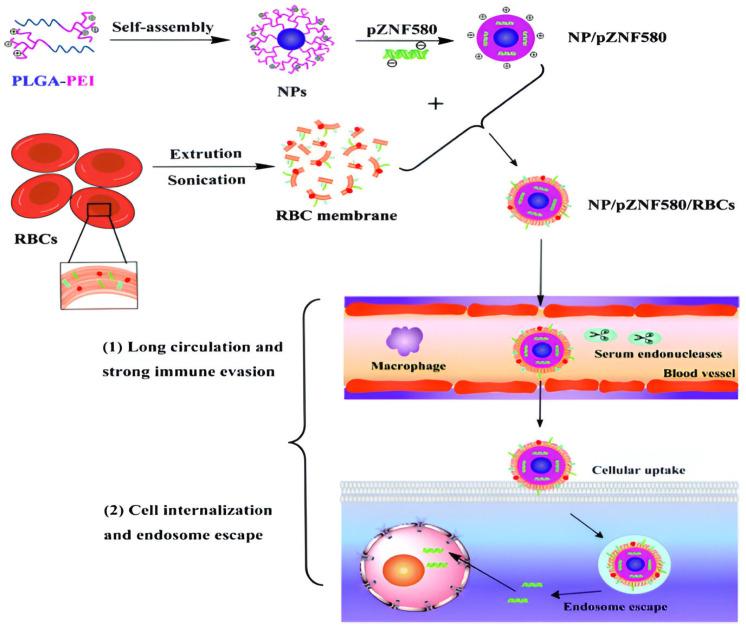
Preparation process of NP/pZNF580/RBCs and their gene delivery by crossing extracellular and intracellular barriers [267].

Receptor-mediated endocytosis facilitates the uptake of CRISPR delivery vectors into early endosomes, progressing to late endosomes and ultimately lysosomes, where the acidic pH and enzymatic activity can degrade CRISPR-Cas9 cargoes. Overcoming this barrier to release cargoes into the cytosol before lysosomal degradation is crucial, and is particularly challenging for non-viral vectors compared to viral vectors. Strategies to achieve endosomal escape include inducing membrane instability through cationic lipids and leveraging the “proton sponge” effect with PEI polymers or any other polymer with a significant secondary and/or tertiary amine character [262]. These approaches aim to enhance the cytosolic delivery of CRISPR components for efficient genome editing. Despite efforts, achieving efficient endosomal release remains challenging. Membrane fusion peptides and cell-penetrating peptides have shown promise in disrupting endosomal/lysosomal membranes, potentially enhancing delivery. Direct cytosolic delivery via nanoparticle membrane fusion bypasses the endosomal pathway, improving cytoplasmic cargo availability. These strategies aim to optimize CRISPR-Cas9 delivery by overcoming endosomal entrapment, which is critical for effective genome editing applications [263,264,265]. Upon cellular uptake, particles often traffic through the endosomal pathway, where they may escape into the cytoplasm to avoid degradation. Successful delivery to the nucleus, where genetic material must ultimately exert its function, involves navigating through nuclear pore complexes or utilizing mechanisms like nuclear localization signals. Unshielded complexes with a positive surface charge facilitate the cellular internalization of nucleic acids, which is crucial for their interaction with intracellular machinery [266]. Hao et al. utilized electrostatic interactions to coat nanoscale red blood cell membranes (RBCM) onto gene complexes, creating a biomimetic delivery system. This approach extended the circulation time and enhanced biocompatibility [267]. Compared to controls, these biomimetic systems reduced phagocytic rates by 52%, highlighting a promising avenue for gene delivery systems employing RBC membrane camouflage. In this study, a biomimetic gene delivery system was developed using amphiphilic polymer PEI (10 kDa)-PLGA-PEI (10 kDa) NPs complexed with the pEGFP-ZNF580 plasmid, coated with the nanoscale RBC membrane via electrostatic interaction, as shown below in Figure 22.

This system showed low cytotoxicity, high transfection efficiency in ECs, enhanced cell migration, and prolonged circulation in vivo, as evidenced by macrophage uptake and in vivo imaging. The RBC membrane-coated gene delivery systems hold promise for effective and biocompatible in vivo applications. To enhance the effectiveness of systemic gene delivery systems, it is crucial to examine how the shell structure imparts stealth qualities to nanoparticles. The shell design, including the use of hydrophilic polymers like PEG and biomimetic elements, is essential for avoiding detection by the reticuloendothelial system (RES) and extending the circulation time. PEGylation and other modifications, such as incorporating erythrocyte membranes or CD47, a “don’t eat me” signal, help reduce immune system detection and clearance. A thorough analysis of these shell designs will provide valuable insights into optimizing gene delivery systems, enhancing their bioavailability, and improving therapeutic efficacy [268].

**Prospects:**

Gene delivery is defined as the transfer of genes into cells to achieve gene expression to accomplish a cure against genetic disorders [269]. Polymers have significant potential in developing non-viral gene delivery systems due to their chemical functional group versatility, tunability, and ability to form various physical architectures. They will be engineered to enhance gene transfer efficiency, biocompatibility, and biodegradability while targeting specific cells or tissues, improving delivery efficiency, reducing off-target effects, and altering immune responses. Despite recent advancements, the limited availability of polymeric materials for drug delivery necessitates the development of novel polymers for specialized targeting and delivery methods. Additionally, pharmaceutical businesses face high clinical trial costs and decreasing success rates for new entities in the R&D pipeline. The complexity of selective targeting, especially in cancer treatments with high toxicity, challenges in clinical translation, high clinical trial expenses, and decreasing success rates highlight the need for more clinical research [270].

One of the primary challenges in using polymers for gene delivery is their cytotoxicity. However, ongoing research is focused on designing and synthesizing new polymers with lower toxicity while maintaining or enhancing their gene delivery efficiency. Strategies such as using biodegradable polymers, controlled polymer architecture, and incorporating biocompatible elements are being explored to mitigate cytotoxicity. Regulatory agencies prioritize therapeutic efficacy and toxicity. Despite biodegradable materials being less harmful, developing effective RNA delivery systems is challenging due to high--throughput screening requirements. Improved discovery efficiency of new non-viral RNA vectors will lead to novel delivery methods addressing unmet therapeutic needs [271]. Non-viral vectors have lower immunotoxicity than viral vectors, and the potential to engineer improved vector performance characteristics, such as size, surface charge, transfection, cytotoxicity, and trafficking, holds promise for future developments [272].

Improving the delivery efficiency of non-viral systems is an essential topic of research. Polymers can be designed to enhance cellular absorption, endosomal escape, and the nuclear localization of genetic material. This can be accomplished by creating smart polymers that respond to specific cellular conditions, incorporating targeting ligands, and developing multifunctional delivery systems that overcome various hurdles to successful gene transport [273].

Natural polymers, such as chitosan, pullulan, and hyaluronic acid, offer biocompatibility and biodegradability, making them attractive candidates for gene delivery. These polymers can be used in their native form or modified to enhance their gene delivery capabilities. The use of natural polymers may also reduce the risk of adverse immune responses and long-term toxicity [173].

Natural polymers can be chemically or physically modified to improve their properties for gene delivery. Modifications may include the introduction of functional groups that enhance DNA binding, the incorporation of stimuli-responsive elements, or the conjugation of targeting moieties. These modifications can improve gene delivery systems’ stability, efficiency, and specificity based on natural polymers [274].

Hybrid systems combining different materials’ strengths are gaining attention in gene delivery research. For instance, nanoparticles coated with polymers can offer enhanced protection and controlled release of genetic material. Similarly, combining man-made polymers with natural polymers can result in systems that leverage the advantages of both types of materials. These hybrid approaches can be designed to optimize biocompatibility, reduce cytotoxicity, and improve overall delivery efficiency [275]. 

## 5. Conclusions

Considerable progress has been made in non-viral gene therapy in recent decades, with advancements in various areas, such as the effectiveness and stability of nucleic acids and the development of new delivery materials. Consequently, researchers have focused on developing novel and efficient non-viral gene vectors. They carefully selected the chemical and physical architecture of the polymeric vectors to enhance the delivery while minimizing toxicity to the host cells. Non-viral gene vectors have shown promising results in transfection, biocompatibility, safety, and stability, outperforming viral gene vectors. Non-viral gene delivery has made significant progress using macromolecules to overcome viral vector limitations, prioritizing safety, efficiency, and targeted delivery. Polymeric materials like PLL, PEI, and PAMAM have shown promise in delivering genetic cargo, while modifications like PLL-g-PEG and star polymers enhance biocompatibility and specificity. Natural polymers and hydrogels offer biodegradable alternatives, but challenges remain in optimizing delivery efficiency and addressing cytotoxicity. Hybrid systems combining nanoparticles with polymers or natural materials represent a promising frontier that needs considerable research and development efforts in the coming years. Future research should prioritize overcoming current challenges, including enhancing gene cargo stability and refining intracellular trafficking. Advancements in macromolecular architecture and judicious selection of functional group chemistry hold promise for realizing safer and more efficient therapeutic interventions for genetic disorders and diseases.

## Figures and Tables

**Table 1:** Structural characteristics and limitations of various polymeric materials used in non-viral gene delivery.

Polymers	Structure	Characteristics	Limitations
**Poly-L-lysine (PLL)**	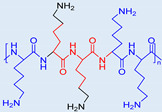	Positively charged, good for DNA binding and delivery	High toxicity, degradable
**Poly(ethylene imine) (PEI)**	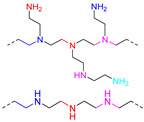	High transfection efficiency, strong DNA binding	High toxicity, non-degradable
**Polyamidoamine (PAMAM)**	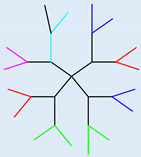	Dendrimer structure, good biocompatibility, uniform size, high degree of branching,	Expensive, potential toxicity at higher generations
**Poly-L-lysine-grafted-polyethylene glycol (PLL-g-PEG)**	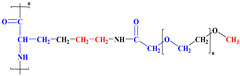	Improved biocompatibility and stability	Complex synthesis, reduced DNA binding capacity
**Hydrogels**	Linear copolymer	Highly absorbent, good for controlled release	Potential for variable degradation rates, aggregation tendency with protein at high pHs
**Natural polymers include chitosan, pullulan, dextrin, and hyaluronic acid.**	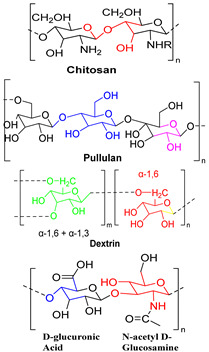	Biodegradable, biocompatible, low toxicity	Limited mechanical properties, batch-to-batch variation, poor solubility in water, low transfection efficiency

**Table 2:** Modification of PAMAM.

Problem Being Solved	Modification	Ref.
**Hydrophobicity-to-lipophilicity ratio**	Alkyl carboxylate, PEG, and cholesteryl chloroformate	[93]
**Improvement of biodegradability**	GLFG oligopeptide, thioketal core	[94,95]
**Prolonged blood circulation half-life and reduced clearance**	PEG attached via a disulfide bond, carboxy betaine acrylamide	[96,97]
**Enhanced cell binding and nucleic acid binding**	Gold nanoparticle core with PAMAM-coated liposomes	[97,98]
**Tissue targeting**	ASSLNIA oligopeptide, monosaccharides (glucose and mannose)	[99,100]

**Table 3:** Capacity and features of gene delivery systems [228].

Vectors	Cargo Capacity	Features	Ref.
**Retrovirus**	7–10 kb	Integrating	[232]
**Adenovirus**	~36 kb	Non-integrating	[233]
**AAV**	4.7 kb	Safely integrating	[228]
**HSV-1 Amplicon**	150 kb	Non-integrating	[234]
**Tol2 Transposon**	~10 kb	Less integrating	[235]
**Episomal**	172–660 kb	Non-integrating	[236]
**Sendai virus**	15,384 kb	Non-integrating	[237,238]
**Linear PEI**	~30 kb	Non-integrating, suitable for transient transfections, and good for plasmid DNA delivery.	[91]
**Branched PEI**	~30 kb	Non-integrating, it has a higher transfection efficiency due to its branched structure, and it is often used for plasmid DNA and RNA delivery.	[239]
